# Integrated metabolomic and transcriptomic analyses elucidate anthocyanin-mediated flesh coloration mechanisms in red-fleshed pear

**DOI:** 10.3389/fpls.2025.1670229

**Published:** 2025-10-31

**Authors:** Mengning Du, Yanan Wang, Xiangzhan Zhang, Suke Wang, Yanli Su, Long Wang, Huabai Xue

**Affiliations:** ^1^ National Key Laboratory for Germplasm Innovation & Utilization of Horticultural Crops, Zhengzhou Fruit Research Institute, Chinese Academy of Agriculture Sciences, Zhengzhou, Henan, China; ^2^ Zhongyuan Research Center, Chinese Academy of Agriculture Sciences, Xinxiang, Henan, China

**Keywords:** pear, red-fleshed, transcriptome and metabolome, anthocyanin, pigmentation

## Abstract

The red-fleshed pears embody a unique and invaluable genetic resource. However, the specific types of metabolites in red-fleshed pears and its molecular mechanisms remain largely unknown. To understand these, targeted carotenoid and anthocyanin metabolomics as well as transcriptomics analyses were carried out in this study, using red-fleshed material ‘M2’ and white-fleshed material ‘XM’. The results showed that the concentrations and proportions of anthocyanins are the predominant determinants of the flesh coloration in red and white cultivars, rather than carotenoids. Notably, cyanidin-3-O-galactoside is the main anthocyanins enriched in red-fleshed pear ‘M2’. Moreover, a total of 6168 common differentially expressed genes (DEGs) between red and white flesh tissues were identified, KEGG and GO enrichment analyses showed that 42 DEGs were significantly enriched in the flavonoid and anthocyanin biosynthesis pathways. Correlation analysis of the transcriptome and metabolome revealed associations between core metabolites and genes, leading to the identification of several key transcription factors potentially involved in anthocyanin biosynthesis, such as *PcWER*, *PcbHLH062*, *PcGSTF12*, and *PcMYB114*. Overall, this study not only provides new insights into the color formation mechanism of red-fleshed pears but also provides guidance for further breeding of red-fleshed pears through the manipulation of anthocyanin biosynthesis.

## Introduction

The pear (*Pyrus* spp.) is a member of the Rosaceae family (Rosaceae), the Pomoideae subfamily (*Pyrus L.*), and the *Pyrus* genus. It is cultivated extensively across the globe and is considered one of the most significant fruit crops. Its cultivation history in China exceeds 3000 years. China is a pivotal center for the origin of the Oriental pear (Wu et al., 2018).The country boasts an exceptionally diverse range of pear species and cultivars, with over 1600 documented cultivars (Xue et al., 2018).

Color is one of the critical indicators for assessing fruit quality, significantly influencing both its aesthetic quality and market value, and is also closely linked to its nutritional value. Among diverse fruits, those exhibiting vibrant colors are often more favored by consumers. Particularly, the red-fleshed pear, a rare wild variant of the European pear (Chagné et al., 2013), its unique flesh coloration renders it potentially competitive in the market. However, research on red-fleshed pears is scarce, particularly concerning the molecular mechanisms underlying the formation of their flesh coloration, which is currently inadequate.

Pigment content is a critical factor determining fruit flesh color, primarily due to two major classes of plant pigments: carotenoids and anthocyanins. Carotenoids are responsible for yellow, orange, and red hues, whereas anthocyanins produce red, purple, and blue tones. Both have been extensively studied in various fruit trees such as apple, grape, and peach (van Nocker et al., 2012; Zhao et al., 2022; Zhong et al., 2022). Nevertheless, in pear, the focus of pigment research differs from that in other fruit trees: on one hand, research on pear flesh color lags far behind that on crops such as apples and kiwifruits (Shu et al., 2023); on the other hand, existing studies on pear pigments mostly focus on the peel, with a severe lack of analysis on the mechanisms of flesh pigment formation.

In research on the regulatory mechanisms of anthocyanin biosynthesis, findings from other fruit trees have provided references for pear studies. Anthocyanin is synthesized through the phenylpropane and flavonoid pathways (Falcone Ferreyra et al., 2012), which involve several key steps. Phenylalanine ammonia-lyase (PAL) converts phenylalanine to cinnamic acid, which is hydroxylated to p-coumaric acid by cinnamate-4-hydroxylase (C4H). 4-coumarate: CoA ligase (4CL) converts p-coumaric acid into p-coumaroyl-CoA. Chalcone synthase (CHS) catalyzes the condensation of p-coumaroyl-CoA with malonyl-CoA to yield naringenin chalcone, which is then isomerized to flavanone by chalcone isomerase (CHI). Subsequent steps involve flavanone 3-hydroxylase (F3H), dihydroflavonol 4-reductase (DFR), and anthocyanidin 3-O-glucosyltransferase (UFGT), culminating in anthocyanin synthesis (Holton and Cornish, 1995; Jaakola, 2013).

In addition, anthocyanin biosynthesis is regulated by various transcription factors (TFs). This process is primarily mediated by the MYB-bHLH-WD40 (MBW) transcription complex (Gonzalez et al., 2008; Yao et al., 2017; Tao et al., 2020). For example, in apples, *MdMYB1* promotes anthocyanin biosynthesis in the peel by binding to the *MdGSTF6* promoter and aiding anthocyanin translocation into vacuoles (Jiang et al., 2019). In red-fleshed apples, a 23 bp repeat sequence in the promoter region of *MYB10* drives self-activation of the gene, promoting anthocyanin accumulation in the flesh (Espley et al., 2007). More recently, research revealed that hormone signaling pathways, such as related to abscisic acid, cytokinin, jasmonic acid, and salicylic acid, also significantly regulate anthocyanin biosynthesis and degradation, further modulating pigmentation in red-fleshed apples (Keller-Przybylkowicz et al., 2024). In kiwifruits, *AcMYB10* interacts with *AcbHLH42* to activate *AcLDOX* and *AcF3GT*, while *AcMADS68* cooperates with *AcMYBF110* and *AcMYB123* to regulate the expression of *AcANS*, collectively promoting anthocyanin biosynthesis (Yu et al., 2019; Liu et al., 2023). In strawberries, the FaMYB5/FaMYB10-FaEGL3-FaLWD1/FaLWD1-like complex positively regulates anthocyanin synthesis (Yue et al., 2023). Mutations in *FaMYB10* lead to differences in flesh color, and the *FaMYB5*-*FaBBX24* module enhances anthocyanin accumulation by upregulating genes such as *F3’H* and *ANR* (Jiang et al., 2023; Zhang et al., 2023). Additionally, anthocyanin biosynthesis is also regulated by some other TFs, including *WRKY*, *NAC*, *ERF*, and *bZIP* (Sun et al., 2023). For peaches, the NAC transcription factor *PpNAP4* coordinates chlorophyll degradation and anthocyanin accumulation by activating chlorophyll catabolism genes (CCGs) and anthocyanin-related genes (Dai et al., 2024).

In pears, most identified genes involved in anthocyanin synthesis have focused on the peel, while fewer studies have addressed flesh biosynthesis. In pear peels, *PyMYB114* forms a regulatory complex with *PyERF3* and *PybHLH3*, which binds to the promoters of *PyDFR*, *PyANS*, and *PyUFGT* to activate downstream genes and promote anthocyanin biosynthesis (Yao et al., 2017). In addition, direct activation of *PpMYB10* by *PpBBX24* can positively regulate light induced anthocyanin accumulation in pear peel (Ou et al., 2020). In pear flesh, current studies have shown that the interaction between *PcERF5* and *PcMYB10* forms the ERF5-MYB10 protein complex, which activates the expression of structural genes and key transcription factor genes (*PcMYB10* and *PcMYB114*) in the anthocyanin biosynthesis pathway, regulating the color of pear flesh (Chang et al., 2023).

Despite these advances, significant gaps remain in the overall understanding of anthocyanin metabolism in red-fleshed pears, particularly in the identification of key metabolites, the synergistic regulation of structural genes and transcription factors, and genetic regulators. Therefore, this study employs integrated metabolomic and transcriptomic analyses to identify the major types of anthocyanins and key candidate genes involved in their biosynthesis in red-fleshed pear flesh, elucidating the regulatory network. This work will not only deepen the understanding of the mechanisms underlying flesh coloration in pears but also provide valuable genetic resources and a theoretical basis for improving fruit color traits through molecular breeding and developing new high-quality red-fleshed pear cultivars.

## Materials and methods

### Plant materials

The study utilized the red-fleshed pear cultivar ‘M2’ and the white-fleshed pear cultivar ‘XM’, both cultivated at the National Fruit Tree Germplasm Zhengzhou Grape and Peach Resource Nursery were used as materials (34.71°N, 113.70°E). This region features a warm temperate continental monsoon climate with four distinct seasons, a mean annual temperature of approximately 16.1°C, an average annual precipitation of 942.3 mm, and sunshine duration of about 2100.3 hours (https://www.zhongyuan.gov.cn/). The experimental orchard is established on moist cinnamon soil with loamy texture, moderate and uniform fertility, good drainage, and a slightly alkaline pH, making it representative of typical pear cultivation areas in the Huang-Huai-Hai region. In this region, the main phenological phases of pear trees typically include budbreak in mid-March, flowering in early April, and a fruit development period from April to July or August, with specific timing varying by cultivar.

Samples were investigated every 10 days starting 20 days after flowering until the fruit was ripening, the samples were taken from the red part of the fruit flesh for ‘M2’ and the same part for ‘XM’. To ensure the reliability and validity of the results, three trees of each cultivar were selected. Five fruits were collected from each tree to serve as biological replicates. All selected trees exhibited consistent growth patterns and good health status, free from visible diseases or pests. All samples were frozen in liquid nitrogen immediately after sampling and stored in -80°C refrigerator.

The samples used for metabolomic and transcriptomic analyses were collected from the same biological replicate fruits at the exact same time point, 40 days after flowering (DAF40). For each cultivar and each biological replicate (n=3), the flesh tissue was immediately divided into two aliquots upon dissection: one was snap-frozen in liquid nitrogen for RNA extraction (transcriptome sequencing), and the other was similarly frozen for metabolite extraction (metabolomic profiling).

### RGB color space

Images of the cross-sections of the fruits were captured in a camera box equipped with a stable light source consisting of a Canon 200DII camera with a 11-55mm STM objective,40cm lightbox and 2 LED monochrome temperature light boards. Utilizing Adobe Photoshop 2022 software, the RGB values of the colored portions of the fruits were analyzed, and five datasets were recorded for each pear cultivar. The CIELAB analysis was conducted using Photoshop, applying the same operational procedures in the Lab color mode.

### Measurement of total anthocyanin content

100 mg of lyophilized powder of pear pulp was weighed and dissolved in 500 μL of extraction solution (methanol with 1% hydrochloric acid); vortexed for 5 min, sonicated for 5 min, centrifuged for 3 min (12, 000 r/min, 4°C), the supernatant was aspirated, and the operation was repeated for one time; two supernatants were combined and transferred to a new tube for anthocyanin measurement (Zhang et al., 2022). Absorbance was measured at 530 and 600 nm using a SpectraMax i3x multimode detection platform (Molecular Devices, Sunny-vale, CA, USA). The anthocyanin content was calculated using the following formula:


$$\text{Total anthocyanins}\left(\text{mg}/\text{g}\right)=\left(A530-A600\right)\times 484.84/\left(m\times 2.35\times 10^{4}\right)$$

Where 484.84 is the molecular weight of cyanidin-3-O-galactoside, 2.35×10^4^ molar extinction coefficient of cyanidin-3-O-galactoside, and m denotes the mass of the flesh.

### Measurement of cyanidin-3-O-galactoside

The samples were ground into a fine powder, and stored at -80°C for subsequent use. 50 mg of the powder was accurately weighed and dissolved in 500 μL of extraction solution (aqueous methanol containing 1% hydrochloric acid). The mixture was vortexed for 5 min, sonicated for 5 min, and centrifuged at 12,000 rpm for 3 min at 4°C. The supernatant was carefully aspirated, and the procedure was repeated once. The combined supernatants were filtered through microporous filter membranes with a 0.22 μm pore size, and stored in a brown injection bottle for HPLC (High Performance Liquid Chromatography) analysis (NY/T 2741-2015).

HPLC analysis was performed using a Waters HPLC system equipped with a C_18_ column (Waters, USA). The column temperature was maintained at 40°C, and the flow rate was set at 0.8 mL/min. The mobile phase consisted of mobile phase A (0.2% formic acid in water) and mobile phase B (100% acetonitrile). The injection volume was 10 μL. A detailed gradient elution program is provided in **Supplementary Table S4**.

### Metabolite data collection and analysis

Metabolomic profiling was conducted using fruit flesh samples collected 40 days after flowering. Sample pretreatment and metabolite assays were conducted at Wuhan Metware Biotechnology Co. Ltd (www.metware.cn) in accordance with standard operating procedures (Di Paola-Naranjo et al., 2004). Samples were freeze-dried, ground into powder, and extracted with 500 μL of methanol/water/hydrochloric acid (500:500:1, V/V/V) per 50 mg of sample (Acevedo de la Cruz et al., 2012). After vortex mixing, ultrasonication, and centrifugation, the supernatant was filtered through a 0.22 μm membrane for LC‐MS/MS (Liquid Chromatography-Tandem Mass Spectrometry) analysis. Analyses were carried out using an UPLC (Ultra Performance Liquid Chromatography) system (ExionLC™ AD, Sciex, USA) coupled with a QTRAP^®^ 6500+ mass spectrometer (Sciex, USA). Chromatographic separation was performed on a Waters ACQUITY BEH C18 column (1.7 µm, 2.1 × 100 mm) with a gradient of 0.1% formic acid in water (mobile phase A) and 0.1% formic acid in methanol (mobile phase B) at a flow rate of 0.35 mL/min. The mass spectrometer was operated in positive ion mode with an ion spray voltage of 5500 V, source temperature of 550 °C, and curtain gas of 35 psi (de Ferrars et al., 2014). Quantification was performed using external standard curves with authenticated reference compounds. Metabolites were identified by matching retention times and multiple reaction monitoring (MRM) transitions with those of the standards (Zhu et al., 2017), and annotations were further validated against the KEGG database. The screening criteria for differential metabolites are FC ≥ 2 or ≤ 0.5 and VIP ≥ 1.

### Transcriptome determination and data analysis

Transcriptome analysis was conducted using fruit flesh samples collected 40 days after flowering, with three biological replicates per cultivar. Total RNA was extracted using a standardized protocol, and RNA integrity was verified using agarose gel electrophoresis and spectrophotometry (NanoDrop). Sequencing libraries were constructed from 1 μg of total RNA per sample using the NEBNext Ultra RNA Library Prep Kit for Illumina (NEB, USA) and sequenced on an Illumina NovaSeq 6000 platform to generate 150 bp paired-end reads. The average sequencing depth was 14× per sample. Clean reads were obtained by processing raw data through fastp (v0.23.2) (Chen et al., 2018), and then aligned to the reference genome PyrusCommunis_BartlettDHv2.0 (https://www.rosaceae.org/species/pyrus/pyrus_communis/genome_v2.0) using HISAT2 (v2.2.1) under default settings (Kim et al., 2015). Gene expression levels were quantified as FPKM values using featureCounts (v2.0.3) (Liao et al., 2014). Differential expression analysis was carried out with DESeq2 (v1.22.1) applying thresholds of |log_2_FC| ≥ 1, FDR < 0.05 and P-Value < 0.05 after Benjamini-Hochberg correction (Love et al., 2014). Functional enrichment analysis of differentially expressed genes for KEGG pathways and GO terms was performed using clusterProfiler (v4.6.0) based on hypergeometric tests.

### Real-time quantitative PCR analysis

The total RNA was isolated using an RNA Extraction Kit (Zoman Biotech, Beijing, China) following the manufacturer’s protocol. Each RNA sample underwent DNase I treatment to eliminate genomic DNA. cDNA was synthesized following the protocol of TransScript One-Step gDNA Removal and cDNA Synthesis SuperMix (TransGen Biotech, Beijing, China). TransStart Top Green qPCR SuperMix (TransGen Biotech, Beijing, China) was utilized for the RT-qPCR analysis, conducted on a Roche LightCycler 480 system (Roche, Basel, Switzerland). The pear *PcTubulin* gene served as the internal control for RT-qPCR analysis. The relative expression levels of the genes were calculated using the 2^−ΔCt^ method. Each gene expression analysis was conducted in triplicate. The specific primers employed in the RT-qPCR analysis are detailed in **Supplementary Table S8**.

### Statistical analysis

Statistical analyses and data visualization were conducted using Microsoft Excel 2019 and GraphPad Prism 9 software. Data are presented as the mean ± SD from three independent replicates. Significance between groups was determined using a two-tailed Student’s t-test. The thresholds for statistical significance were defined as follows: not significant (ns) for *p* > 0.05; * for *p* ≤ 0.05; ** for *p* ≤ 0.01; *** for *p* ≤ 0.001; and **** for *p* ≤ 0.0001.

## Results

### Color conversion during fruit development

**Figure 1:**
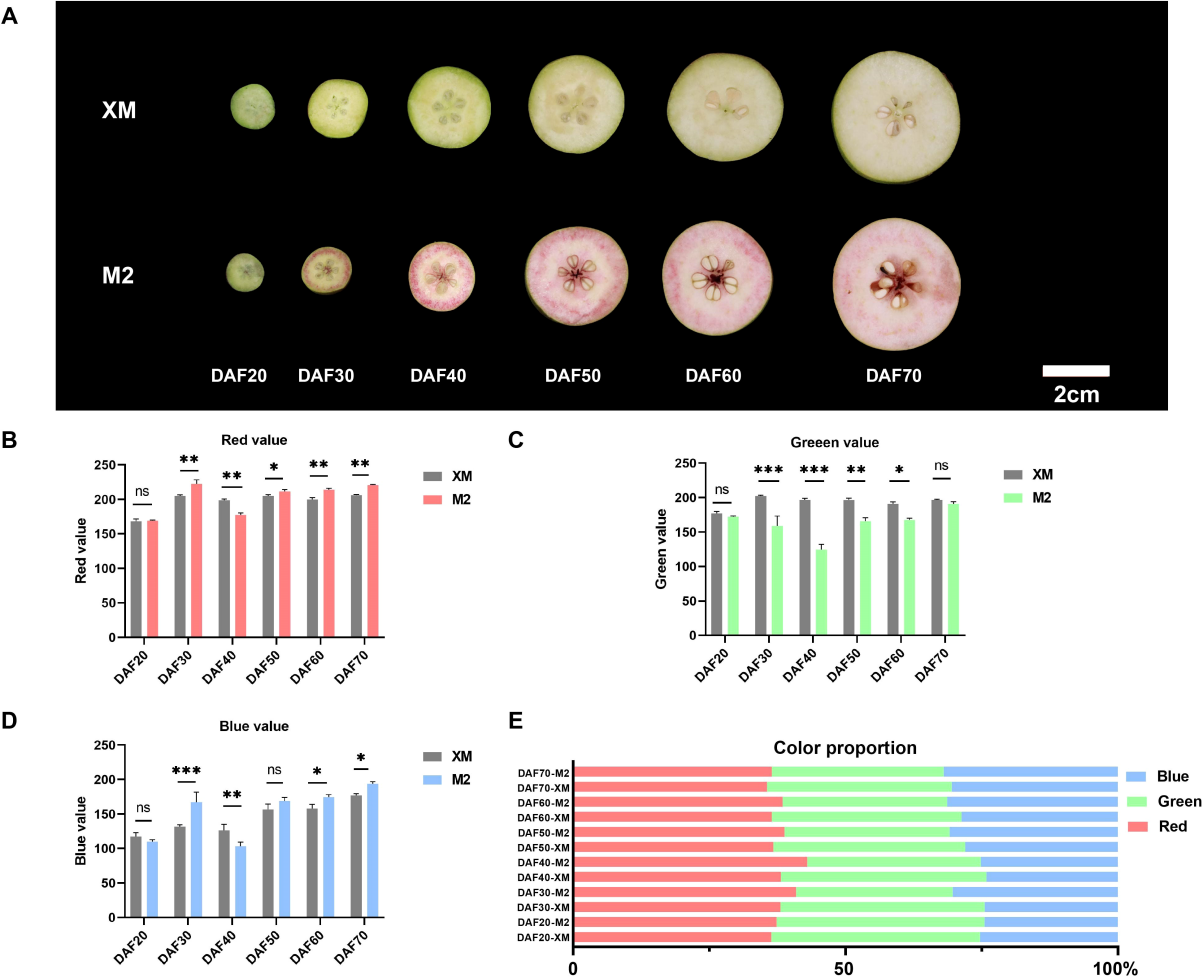
Morphology and characteristics of coloring of white-flesh cultivar ‘XM’ and red-fleshed cultivar ‘M2’ **(A)** Morphological observation from DAF20 to DAF70. **(B)** Red value. **(C)** Green value. **(D)** Blue value. **(E)** Color proportion. ns, not significant, for *p* > 0.05; * for *p* ≤ 0.05; ** for *p* ≤ 0.01; *** for *p* ≤ 0.001; and **** for *p* ≤ 0.0001.

In this study, two of the genetic resources, ‘M2’characterized by its red flesh and white-fleshed ‘XM’, are both European pears with different flesh color. To explore the mechanism of flesh color conversion, we divided the process into 6 stages, spanning from 20 days after flowering (DAF20) throughout the entire growth and development period until ripening (DAF70). The flesh color on the cut surfaces was systematically photographed and documented every 10 days (**Figure 1A**). Initially, the flesh of ‘M2’ did not exhibit a red coloration. At DAF20, both ‘M2’ and ‘XM’ displayed a light green flesh during the early fruit stage. By DAF30, ‘M2’ developed a ring of red coloration near the rind, with a slight reddish hue in the core and partially retained light green in flesh. By DAF40, the flesh of ‘M2’ no longer had a distinct green appearance; it turned bright red with evident annular red flesh near the peel. Throughout subsequent maturation, the flesh color of ‘M2’ transitioned from bright red to pink while losing its distinct border of annular red tissue. Concurrently, the green pigmentation in ‘XM’ faded away, exhibiting typical characteristics observed in white-fleshed pears.

Analysis of photographs taken at each developmental stage using the RGB color space revealed changes in fruit phenotype and variations in the intensity of the red (R), green (G), and blue (B) components. The red intensity and blue intensity in ‘M2’ were higher than in ‘XM’ in most periods (**Figures 1B, D**), while ‘XM’ exhibited a higher green intensity than ‘M2’ (*p* < 0.05; **Figure 1C**), with red intensity peaking at DAF40. Moreover, from DAF30 onward, the R value in ‘M2’ was consistently greater than both the G and B values, demonstrating a sustained and prominent red pigmentation. Analysis of RGB proportion further confirmed these trends: during the mid-development stages, the red component dominated in ‘M2’, reaching up to 43% of the total RGB by DAF40. In contrast, the green component remained predominant in ‘XM’ during early fruit development, representing 38% of the total at DAF20 (**Figure 1E**). These quantitative outcomes robustly support the observed phenotypic color transitions.

In addition to the RGB color space, the CIELAB color space is widely adopted in plant color research. Although direct measurement using a colorimeter was not feasible due to the nature of our experimental samples, we have additionally provided CIELAB values (L*, a*, b*) converted from RGB data using Photoshop, following the same procedure as applied for the RGB color analysis (**Supplementary Figure S6**). In the CIELAB analysis, both the a* and L* values of ‘M2’ were significantly higher than those of ‘XM’ at DAF40 (*p* < 0.001), which is consistent with the RGB results, indicating that ‘M2’ had the deepest coloration at this stage. Meanwhile, the ΔE values were relatively high during both the DAF20-30 and DAF40-50 periods, which also aligns with our phenotypic observations (**Figure 1A**), confirming that noticeable coloring and fading occurred during these two phases.

### Identification and quantification of metabolites in pear flesh

**Figure 2:**
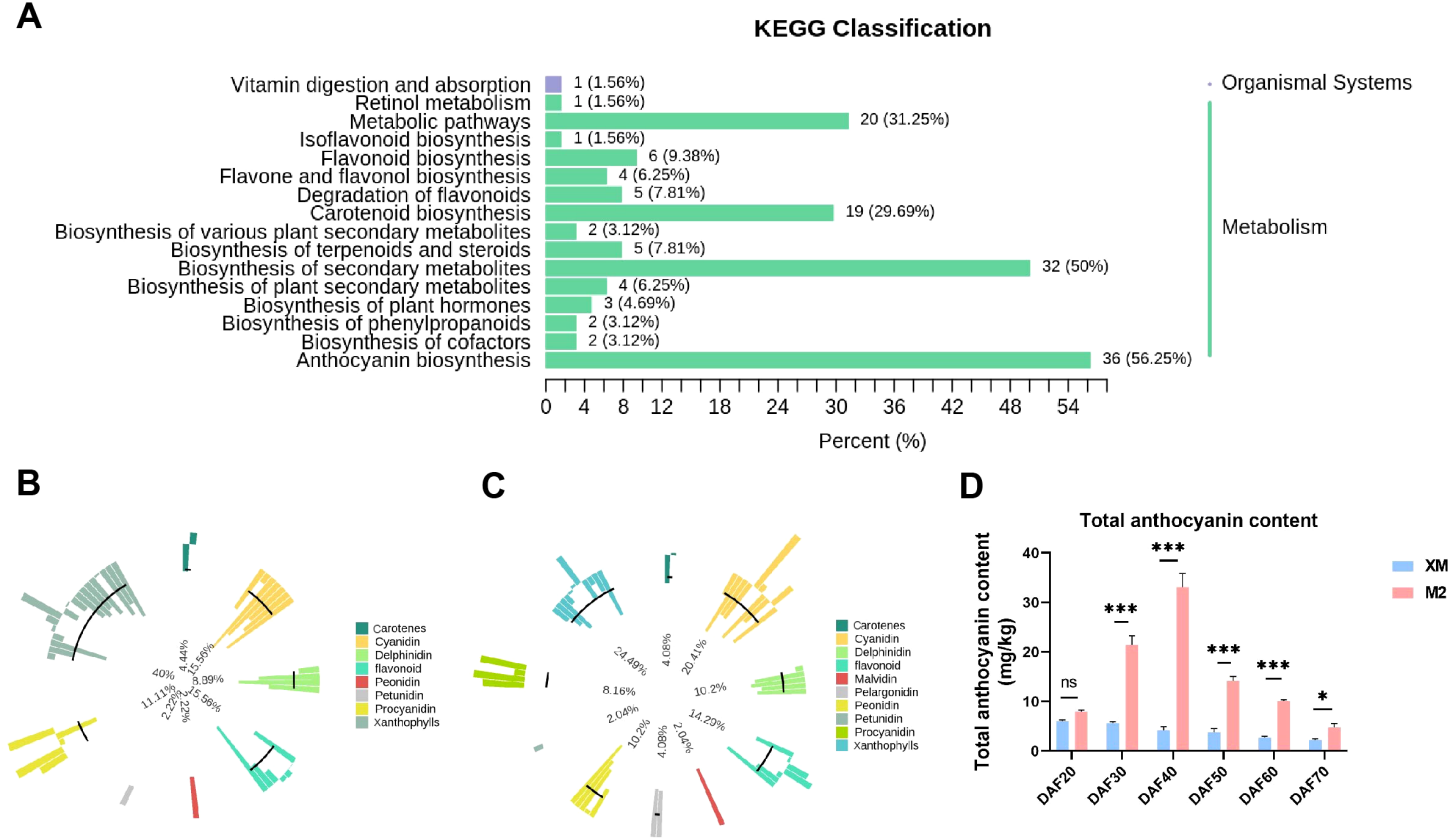
Analysis of metabolome results. **(A)** KEGG enrichment analysis of DEMs. **(B)** Metabolite primary classification proportion circle chart of ‘XM’. The outermost circle of the graph shows the substances of different categories and their relative contents, one color represents one type of substance, and the length of the column represents the relative content of the substance; the second circle counting from the outside with the length of the line segment represents the proportion of the classified substances to the total number of all substances; the more substances under the classification, the longer the line segment; the innermost circle is the ratio of the number of each type of substance to the number of all substances. **(C)** Metabolite primary classification proportion circle chart of ‘M2’. **(D)** Total anthocyanin content. not significant (ns) for *p* > 0.05; * for *p* ≤ 0.05 and *** for *p* ≤ 0.001.

It is reported that the pigments in colored fruits are composed of carotenoids and anthocyanins (Yoo et al., 2017; Guo et al., 2021; Diao et al., 2023; Ro et al., 2024). To understand what pigment contribute to the color changes of two pear cultivars, anthocyanin-targeted and carotenoid-targeted metabolome were performed in the flesh of DAF40, a key stage characterized by visually apparent coloration changes (**Figure 1A**). A total of 108 anthocyanins and 68 carotenoids were identified from the metabolome (**Supplementary Table S1**), and their enrichment was analyzed using KEGG (**Figure 2A**). The relative content of metabolites and their proportion of the total metabolites accounted for by the categorized substances indicated that anthocyanins were the primary metabolites responsible for the color difference between red and white flesh, rather than carotenoids (**Figures 2B, C**).

To substantiate our initial findings and elucidate the variations in anthocyanin content across various stages of maturity, we quantified the anthocyanin levels in samples collected at distinct developmental phases (**Figure 2D**). Encouragingly, the data obtained from these follow-up experiments exhibit a high degree of concordance with the metabolomic analysis outcomes, thereby reaffirming the pivotal role of anthocyanins in modulating fruit coloration. Moreover, a gradual decline in anthocyanin content was observed throughout the developmental stages, correlating with the observed phenotypic changes. This suggests that the flesh color of ‘M2’ shifted from colorless to pigmented and then experienced a progressive loss of pigmentation (**Figure 2D**).

**Figure 3:**
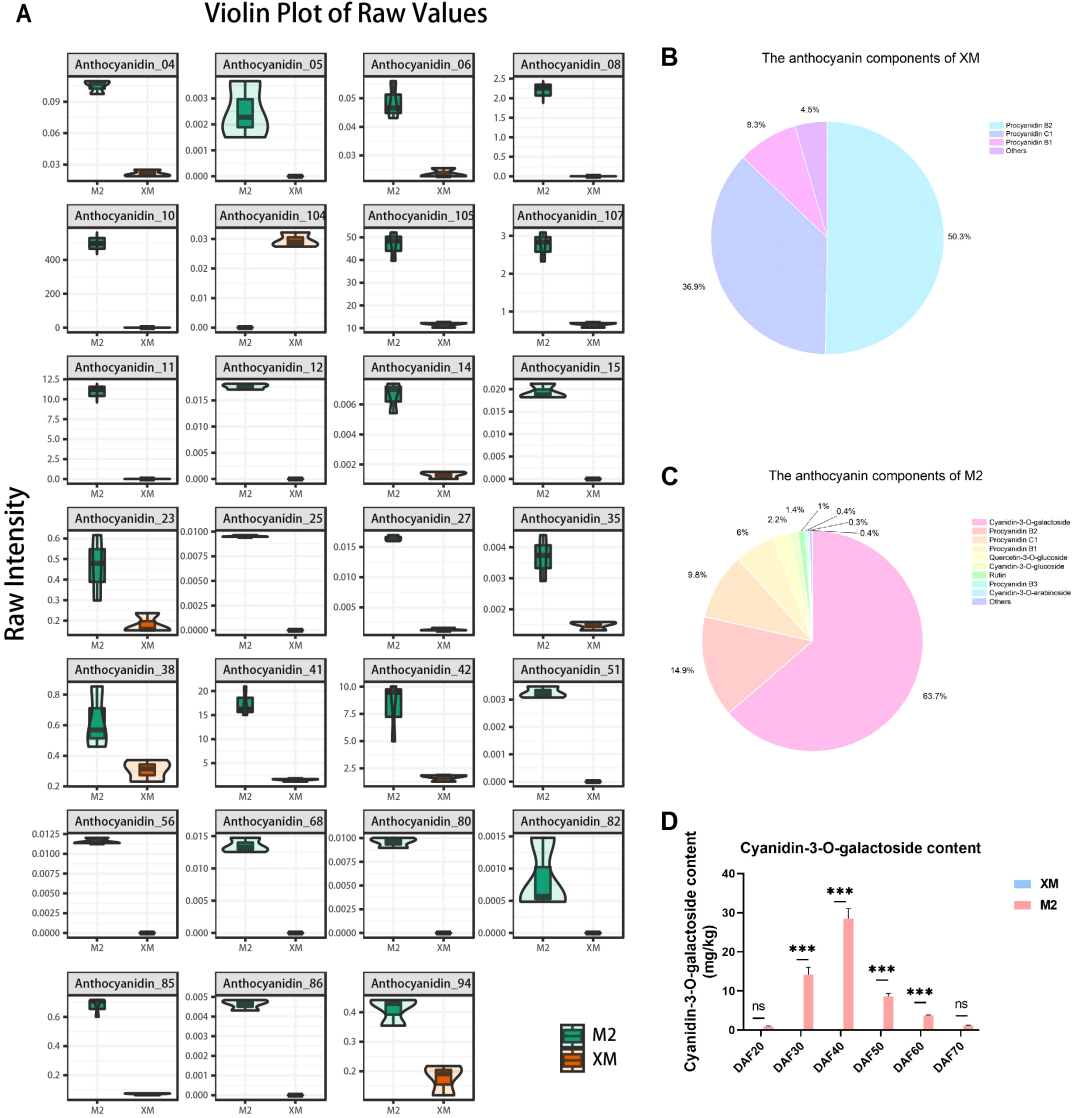
Anthocyanin components analysis. **(A)** Differential metabolism anthocyanin violin plot. **(B)** Anthocyanin fraction pie chart of ‘XM’. **(C)** Anthocyanin fraction pie chart of ‘M2’. **(D)** Cyanidin-3-galactoside content. not significant (ns) for *p* > 0.05 and *** for *p* ≤ 0.001.

Next, a detailed analysis of anthocyanin composition was conducted. Among the 27 differentially metabolized anthocyanin species, 26 showed higher accumulation in red flesh (**Figure 3A**). This finding suggests that differences in anthocyanin composition are responsible for the observed color differences between red and white flesh. The total anthocyanin content in ‘M2’ was 5.64-fold higher than that in the white-flesh ‘XM’ (**Supplementary Table S1**). The three predominant anthocyanidins in ‘XM’ were colorless proanthocyanidins, accounting for 95.49% of its total anthocyanidin content (**Figure 3B**), indicating minimal anthocyanidin pigmentation in ‘XM’. In contrast, the three most abundant anthocyanidins in ‘M2’ were cyanidin-3-O-galactoside, proanthocyanidin B2, and proanthocyanidin C1, collectively representing 88.29% of its total anthocyanins content (**Figure 3C**). Proanthocyanidin, being colorless, and cyanidin-3-O-galactoside, which constituted 63.67% of the total anthocyanidins, were key components in ‘XM’ and ‘M2’, respectively. In summary, cyanidin-3-O-galactoside was identified as the major pigment responsible for the red coloration in ‘M2’.

In order to further investigate the changes in cyanidin-3-O-galactoside content across various developmental stages, we quantified the cyanidin-3-O-galactoside levels in the flesh across 6 distinct developmental stages, and found that the cyanidin-3-O-galactoside content was significantly higher (*p* < 0.01) in ‘M2’ than in ‘XM’ at every developmental stage, with the difference being most pronounced at DAF40 (**Figure 3D**). Identification of anthocyanin fractions at each developmental stage using HPLC revealed that the only highest peak in the chromatogram was cyanidin-3-O-galactoside (**Supplementary Figure S2**).

Additionally, the comparison between cyanidin-3-O-galactoside content (**Figure 3D**) and total anthocyanins (**Figure 2D**) showed a similar trend and equivalent levels. Furthermore, cyanidin-3-O-galactoside content was marginally lower than the total anthocyanin content, representing up to 85% of the total at its peak accumulation (DAF40) This finding further supports the notion that cyanidin-3-O-galactoside is a predominant pigment in the red flesh of pears. This concordance not only bolsters the credibility of the metabolomic analysis but also lays a robust foundation for a comprehensive understanding of the dynamic fluctuations in anthocyanin levels throughout the fruit ripening process.

### Transcriptome analysis of white and red flesh in pears

To explore the molecular mechanism of anthocyanin biosynthesis, high-throughput RNA-Seq analysis of ‘M2’ and ‘XM’ fruit flesh on DAF40 was performed. A total of 40.91 Gb raw reads with sequencing error rate lower than 0.03% were obtained from the sequencing of all 6 libraries, and the biological replicates of different samples were clustered together to show high reproducibility (**Supplementary Figure S1A**). A total of 28.30 Gb clean reads were obtained with Q20 > 98%, Q30 > 94.15% after filtering and the GC content ranged from 47.13% to 47.39% (**Supplementary Table S2**). More than 90% of the clean reads from ‘M2’ and ‘XM’ could be unique mapped to reference genome (**Supplementary Table S3**). Totally, 6168 differentially expressed genes (DEGs) were shared among two fruit flesh, among these 3090 were down-regulated in ‘M2’ and 3078 were up-regulated (**Supplementary Figure S1B**).

**Figure 4:**
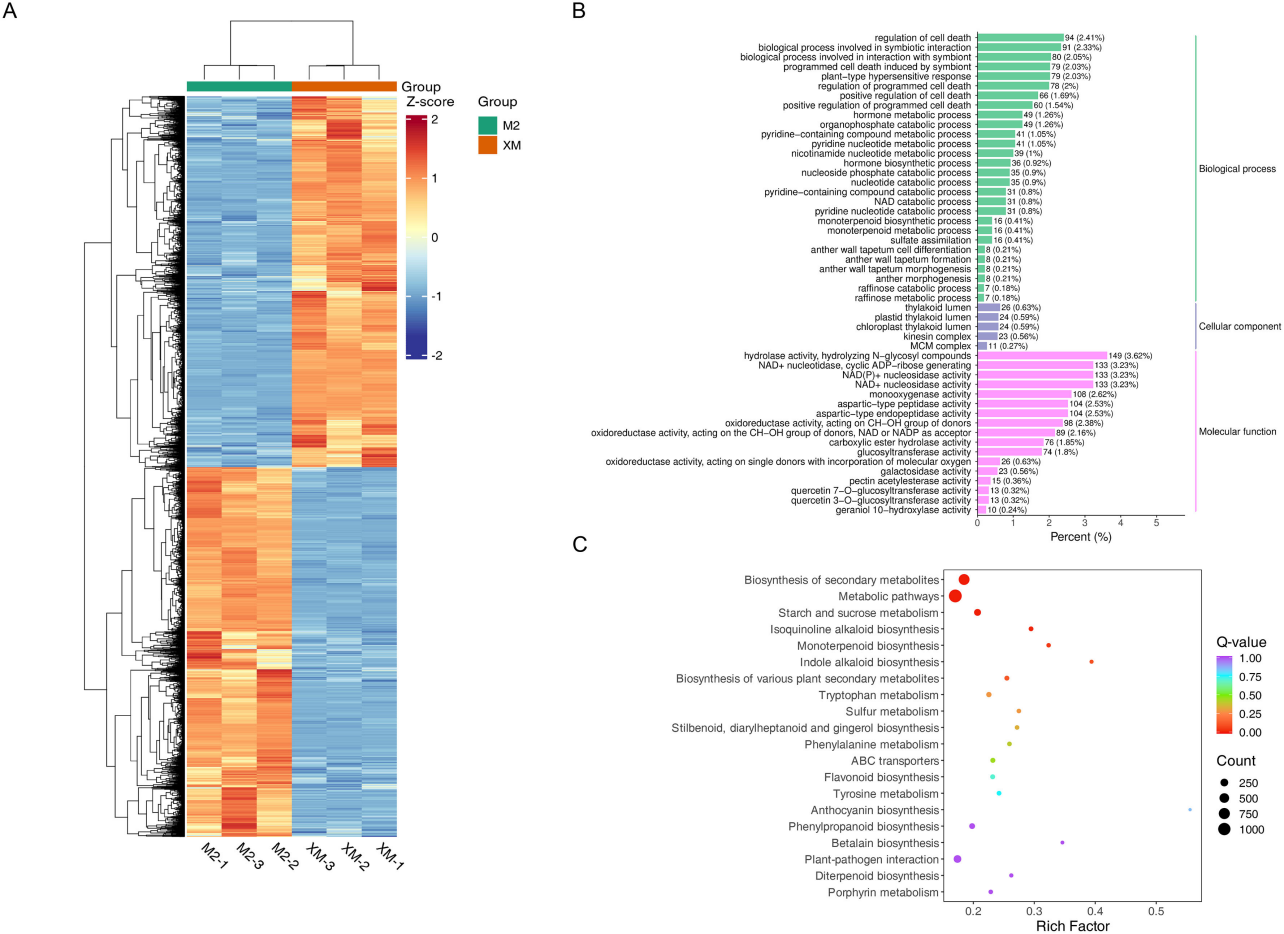
Differential gene expression. **(A)** Heat map of differentially expressed gene clusters. **(B)** Bar graph of differential gene GO enrichment. **(C)** Scatter plot of differential gene KEGG enrichment.

To further explore the function of these DEGs, Gene Ontology (GO) and Kyoto Encyclopedia of Genes and Genomes (KEGG) enrichment analyses were performed. (**Figures 4B**, **C**). The GO enrichment analysis revealed significant enrichment in molecular functions related to nucleotidase activity, NAD(P)+ nucleotidase activity, NAD+ nucleotidase activity, hydrolase activity, and N-glycosyl compound hydrolysis in red flesh compared to white flesh. These functions are integral to various metabolic processes and suggest a role in the differential pigmentation observed (**Figure 4B**). The KEGG analysis mapped the DEGs to 138 metabolic pathways, with significant enrichment observed in pathways such as secondary metabolite biosynthesis, starch and sucrose metabolism, flavonoid biosynthesis, and anthocyanin biosynthesis. These pathways are particularly relevant to the pigmentation differences, as they include key steps in the production and regulation of anthocyanins, the pigments responsible for the red coloration in ‘M2’.

Through transcriptome analysis, we also identified 15 DEGs with significant fold differences, among which *PcGSTF12* (*Pycom17g27080*) and *PcMYB114* (*Pycom05g25770*) may be related to anthocyanin biosynthesis (**Supplementary Figure S3**).

### Analysis of anthocyanin biosynthetic genes

**Figure 5:**
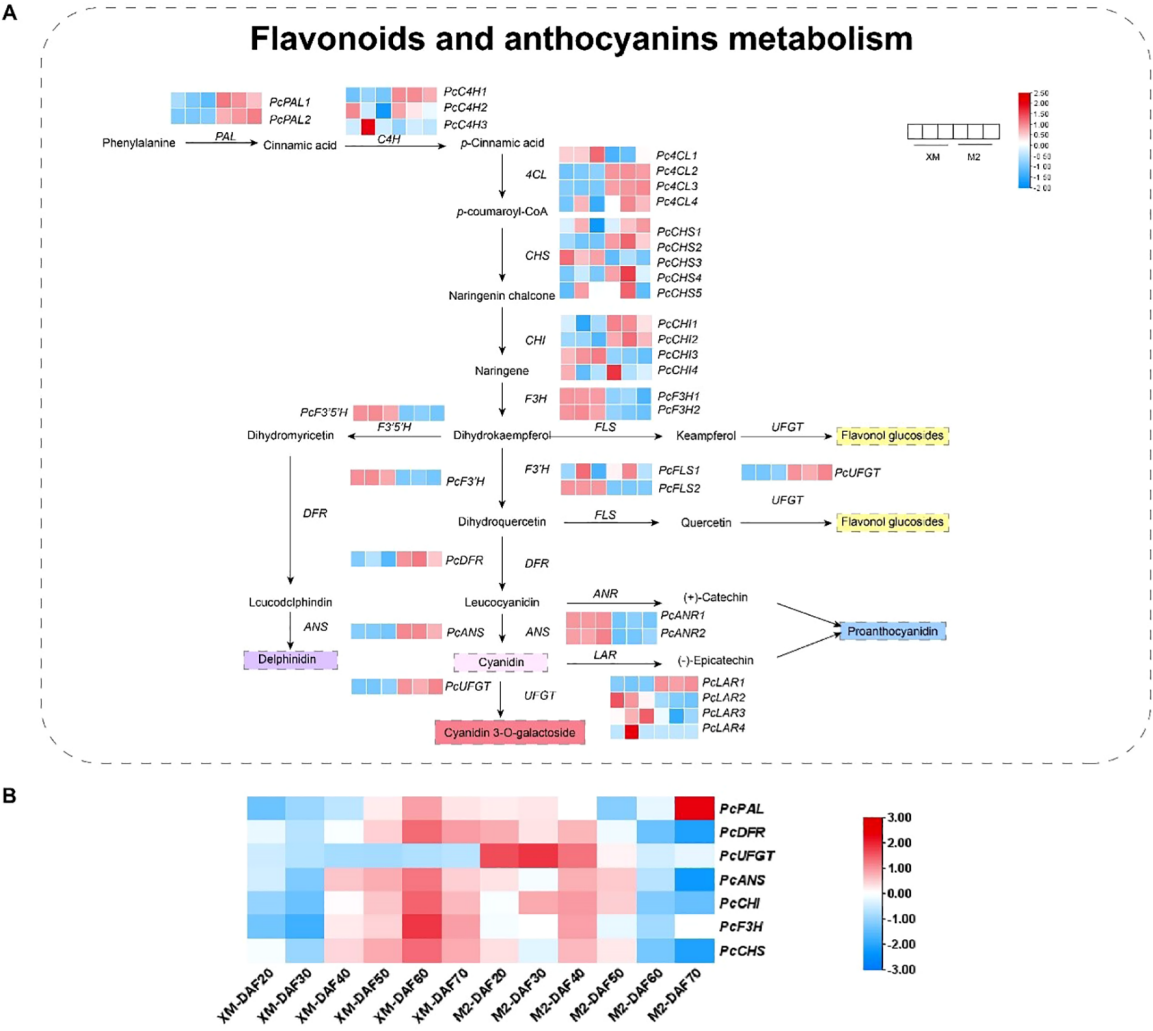
Combined analysis of metabolites and genes involved in flavonoid and anthocyanin biosynthesis. **(A)** Flavonoid and anthocyanin biosynthesis metabolites versus RNA-seq FPKM values. **(B)** Relative expression of structural genes.

The biosynthesis of anthocyanins, which are responsible for the red pigmentation in ‘M2’, is a complex process regulated by multiple structural genes. To explore the biosynthesis of anthocyanins, a total of 38 structural genes associated with the anthocyanin biosynthesis pathway were identified in the transcriptome data (**Figure 5A**; **Supplementary Table S7**), including key enzymes such as phenylalanine ammonia-lyase (PAL), cinnamic acid 4-hydroxylase (C4H), 4-coumaric acid (4CL), chalcone isomerase (CHI), chalcone synthase (CHS), flavanone 3-hydroxylase (F3H), flavonoid 3’-hydroxylase (F3’H), dihydroflavonol 4-reductase (DFR), anthocyanidin synthase (ANS), UDP-glycose flavonoid glycosyl transferase (UFGT), flavonoid synthase (FLS), flavonoid 3’,5’-hydroxylase (F3’5’H), leucoanthocyanin reductase (LAR), and anthocyanidin reductase (ANR).These genes are known to play pivotal roles in the anthocyanin biosynthesis pathway, and their expression patterns can significantly influence the accumulation of anthocyanins in fruit tissues. In contrast, expression levels of carotenoid biosynthetic genes remained low and showed no significant differential expression between cultivars (**Supplementary Table S9**), further supporting that carotenoids are not associated with the flesh coloration trait.

The expression analysis revealed significant up-regulation of genes such as *PcPALs*, *PcC4Hs*, *Pc4CLs*, *PcCHSs*, and *PcCHIs* in the red flesh during the early stages of anthocyanin synthesis, indicating their potential involvement in the initiation of pigment production. Notably, within the *Pc4CLs*, *PcCHSs*, and *PcCHIs*, different transcript levels suggest diverse functional roles within the PAL pathway. During the mid-stage of anthocyanin synthesis, genes like *PcF3Hs*, *PcF3’5’Hs*, and *PcF3’Hs* were significantly up-regulated in white flesh, highlighting a differential expression pattern between the two fruit types. In the late stages, the expression of genes involved in the synthesis of colorless proanthocyanidins, such as *PcANRs*, was significantly higher in white flesh, while the expression of genes associated with the synthesis of colored anthocyanidins, including *PcDFRs*, *PcANSs*, and *PcUFGTs*, was markedly up-regulated in red flesh (**Figure 5A**; **Supplementary Table S7**). This suggests a specific role for these genes in the production of colored anthocyanins in ‘M2’.

The quantitative verification of structural gene expression related to anthocyanin synthesis at different developmental stages confirmed that genes like *PcDFR*, *PcUFGT*, *PcANS*, *PcCHI*, *PcF3H*, and *PcCHS* were up-regulated in ‘M2’ during the early and middle stages of fruit development (**Figure 5B**), aligning with the observed increase in anthocyanin accumulation during these periods. Intriguingly, *PcUFGT* was down-regulated in white flesh across all periods, which may explain the inability of ‘XM’ to synthesize colored anthocyanins.

### Integrated analysis of the transcriptome and metabolome and identification of TFs

The biosynthesis of anthocyanins is under tight transcriptional control, with various transcription factors (TFs) playing crucial roles in regulating anthocyanin production across different species. Among the DEGs, a total of 79 TFs were identified, encompassing families such as *MYBs*, *bHLHs*, *NACs*, *AP2/ERFs*, *WRKYs*, and *MADSs* (**Supplementary Table S5**). These TFs are known to orchestrate the expression of structural genes involved in anthocyanin biosynthesis.

**Figure 6:**
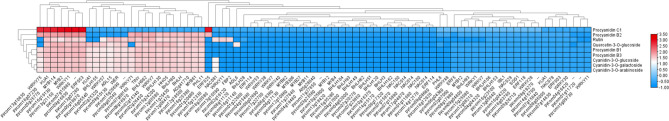
Heat map of correlation analysis.

To further investigate the potential regulatory interactions between these TFs and anthocyanin-related metabolites, a correlation analysis between the TFs and the first nine differentially accumulated metabolites was conducted. The results revealed that nine metabolites exhibited strong correlations with these 24 TFs, with at least one of the correlations having an R-value greater than 0.80 (**Figure 6**). Notably, TFs such as *MYB114*, *bHLH130*, *bHLH062*, *WRKY71*, *WRKY11*, and *ERF053* showed significant associations and were significantly correlated with the abundance of Cyanidin-3-O-galactoside (**Supplementary Table S5**), suggesting their potential roles in the regulation of anthocyanin biosynthesis in red-fleshed pears.

### RT-qPCR analysis of candidate genes

**Figure 7:**
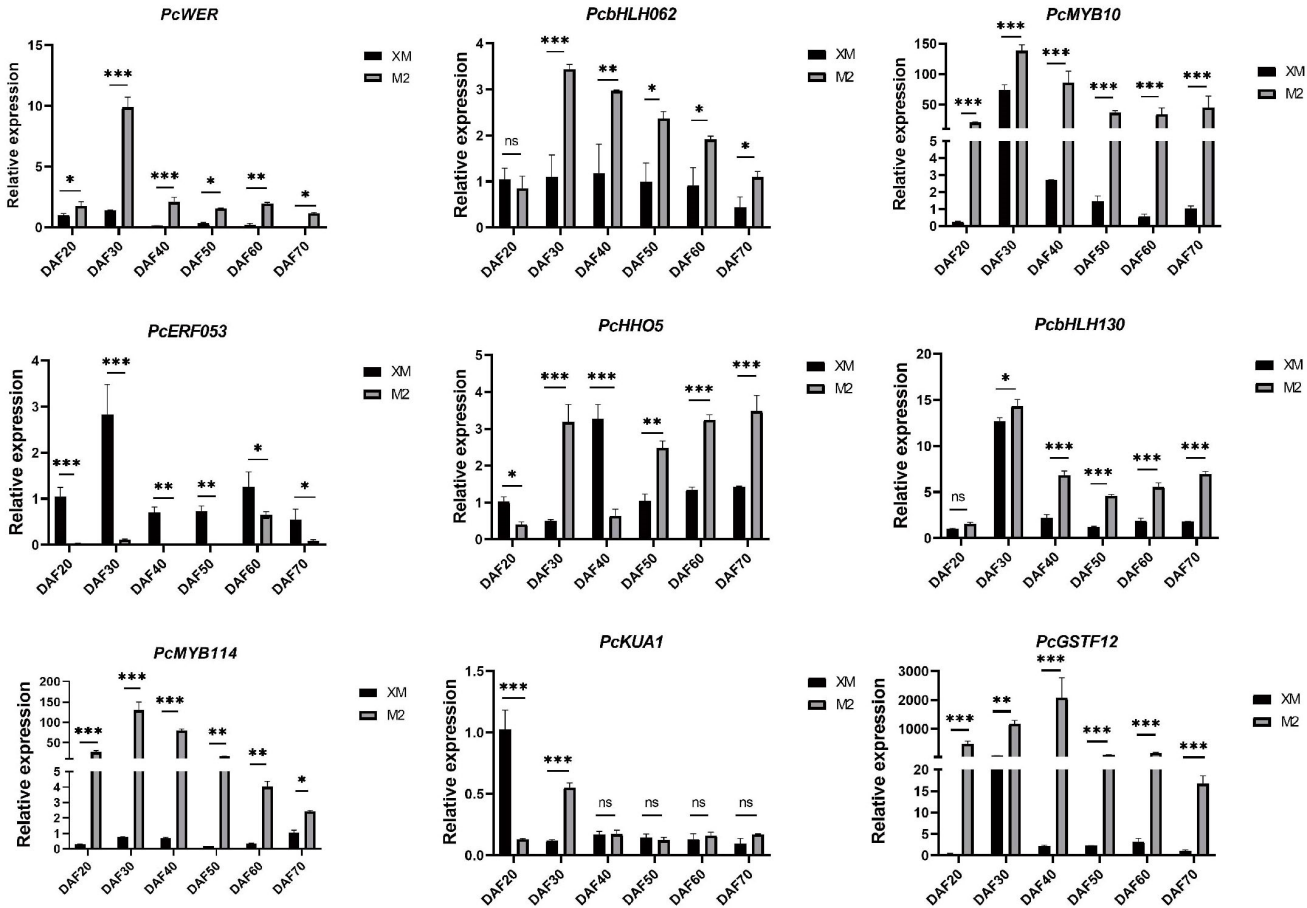
RT-qPCR analysis of candidate genes. ns, not significant, for *p* > 0.05; * for *p* ≤ 0.05; ** for *p* ≤ 0.01; *** for *p* ≤ 0.001; and **** for *p* ≤ 0.0001.

To further screen structural genes and TFs, we conducted RT-qPCR analysis of a panel of candidate genes associated with anthocyanin biosynthesis, including *PcWER*, *PcHHO5*, *PcKUA1*, *PcbHLH130*, *PcbHLH062*, *PcERF053*, *PcGSTF12*, and *PcMYB114*, based on transcriptome and metabolome data (**Figure 7**).

The RT-qPCR results confirmed that the expression levels of *PcWER*, *PcbHLH062*, *PcGSTF12*, and *PcMYB114* were significantly higher in the red flesh compared to those with the white flesh. Notably, the expression profiles of *PcWER*, *PcGSTF12*, and *PcMYB114* exhibited a positive correlation with the quantified anthocyanin content, thereby implying a potential regulatory function in the biosynthesis of anthocyanin. This observed up-regulation in red flesh tissues indicates that these genes may serve as key regulators in the induction of anthocyanin synthesis pathways.

## Discussion

Red-fleshed pears represent a rare trait predominantly found in limited European germplasms, with the molecular mechanisms remaining poorly characterized. To address this knowledge gap, we conducted an integrated transcriptomic and metabolomic analysis of red-fleshed pear fruit, revealing novel mechanistic insights with implications for both basic research and pear breeding.

A pronounced difference in anthocyanin content was observed between red- and white-fleshed cultivars (**Figure 3**), confirming the central role of anthocyanin accumulation in flesh coloration. Metabonomic profiling identified cyanidin-3-galactoside as the predominant anthocyanin which consistent with earlier reports in pear (Chang et al., 2023). While anthocyanin-mediated pigmentation is widespread among horticultural crops, the composition in pear flesh is notably less diverse compared to that in peel tissues (Ngo and Zhao, 2009; Zhai et al., 2016). Cross-species comparisons reveal that although cyanidin derivatives are common, for instance, cyanidin-3-glucoside in red-fleshed peaches (Ding et al., 2020) and both cyanidin-3-O-glucoside and cyanidin-3-galactoside in red-fleshed apples (van Nocker et al., 2012; Shi et al., 2022) and strawberries prioritize pelargonidin-3-glucoside (Kelebek and Selli, 2011). And pear flesh exhibits remarkable anthocyanin component singularity (only cyanidin-3-galactoside as the major pigment). This singularity simplifies the dissection of regulatory mechanisms, as it eliminates confounding effects from multiple anthocyanin biosynthesis branches, and provides an advantage for targeted genetic manipulation in pear breeding.

Notably, ‘M2’ flesh color exhibits a dynamic trajectory: it intensifies to bright red in early development, then fades to pink as maturity approaches (**Figure 1A**), accompanied by a significant decline in anthocyanin content (**Figure 2D**). This phenotype is distinct from most red-fleshed fruits (e.g., apples, peaches) where anthocyanin content increases or stabilizes during ripening (Chagné et al., 2013; Ding et al., 2020), which is an under-explored facet in current research.

Anthocyanin biosynthesis in ‘M2’ flesh is tightly linked to the expression of core structural genes. During early fruit development, key structural genes, including *PcCHS*, *PcCHI*, *PcF3H*, *PcANS*, *PcDFR*, and *PcUFGT*, were highly expressed during early developmental stages when coloration was most intense(**Figure 5B**), indicating their crucial roles in pigmentation during the early stages of development. Among these, *PcDFR* (catalyzing dihydroflavonol reduction) and *PcANS* (converting flavonols to anthocyanidins) are critical for directing metabolic flux toward anthocyanin synthesis. *PcUFGT*, which converts flavonols into anthocyanins, shows high expression in red-fleshed samples, potentially leading to increased anthocyanin accumulation (Keller-Przybylkowicz et al., 2024). The significant differential expression of *PcUFGT* between red and white flesh may represent a critical step in determining flesh color, specifically through the synthesis of the colored anthocyanin cyanidin-3-galactoside. Anthocyanins are synthesized on the cytoplasmic surface of the endoplasmic reticulum and then transported to the vesicles for anthocyanin accumulation (Marrs et al., 1995). Gene families related to anthocyanin transport, such as the glutathione S-transferase (GST) family, were also identified in the DEGs of this study. Notably, the expression pattern of *PcGSTF12* mirrored that of anthocyanin content, with a decrease in relative expression in the later stages of fruit development. This suggests a concomitant decline in the rate of anthocyanin transport during fruit maturation.

Anthocyanin biosynthesis is also regulated transcriptionally by TFs, with the MBW complex (MYB-bHLH-WD repeat) being a key regulator (Xu et al., 2014; Wang et al., 2015; Li et al., 2020a; Liu et al., 2021). R2R3-MYB TFs are well-studied for their roles in various plant (Niu et al., 2010; Petroni and Tonelli, 2011; Albert, 2015; Zhang et al., 2017; Lai et al., 2019; Yuan et al., 2020). Certain bHLH TFs also positively regulate anthocyanin biosynthesis (Li et al., 2016, 2020b; Deng et al., 2021). Our transcriptome-metabolome correlation analysis identified four TFs with strong associations to anthocyanin metabolites: *PcWER* (R2R3-MYB), *PcbHLH062* (bHLH), and *PcMYB114* (R2R3-MYB) showed positive correlations, while *PcERF053* (ERF) exhibited negative regulation (**Figure 7**). RT-qPCR validation confirmed their differential expression, supporting their roles as core regulators. Notably, *PcWER*’s regulatory role in pear flesh is a novel finding: in apple peel, *MdWER* may form a protein complex with *MdERF109* to promote anthocyanin accumulation (Zhang et al., 2024). Interestingly, some candidate genes (e.g., *PcGSTF12*) previously linked to peel pigmentation (Li et al., 2022) also emerged in our flesh-specific analysis, suggesting shared regulatory mechanisms or a lack of tissue specificity in anthocyanin regulation.

In addition to the endogenous regulation of structural genes and transcription factors related to anthocyanin biosynthesis, exogenous environmental factors can also lead to anthocyanin degradation. As previously discussed, anthocyanin synthesis is also modulated by environmental factors, with light being a significant influence (Takos et al., 2006). Typically, light promotes anthocyanin synthesis; however, under conditions of low light intensity or darkness, the activity of the MBW complex is reduced the activity of the MBW complex is reduced, resulting in the down-regulation of structural genes and a subsequent decrease in anthocyanin content (Chalker-Scott, 1999; Takos et al., 2006). However, for Zhengzhou, China, where our material was grown, the fruits developed in spring and early summer, and light intensity increased, thus, light is unlikely to be the primary cause for the discoloration of ‘M2’.

Previous studies have indicated that high temperatures could reduce anthocyanin accumulation in a cultivar of plant species, including apples (Lin-Wang et al., 2011), grapes (de Rosas et al., 2017) and potatoes (Han et al., 2024), Based on previous studies (Chen et al., 2021), we hypothesized that the decline in anthocyanin levels might be caused by the increase in temperature at the later stage of fruit development. Therefore, during each survey, we recorded the daily temperature (**Supplementary Figure S4**). The temperature in the planting area showed a marked rise at DAF50, while the anthocyanin content of ‘M2’ decreased significantly during this stage. This suggests a probable relationship between anthocyanins and temperature. Recent studies on pears have also shown that high temperatures have a negative regulatory effect on anthocyanins (Wang et al., 2025). It enhances our confidence in the idea that the fading of red-fleshed pear fruit is related to high temperature. The quantitative expression analysis of structural genes associated with anthocyanin biosynthesis (**Figure 5B**), reveals a notable decline in transcript levels after day 50 of fruit development (DAF50). This decrease in gene expression correlates with the observed reduction in anthocyanin synthesis during the later stages of fruit maturation, aligning with fluctuations in local temperature patterns. Additionally, as the fruit develops and grows, the fruit size correspondingly increases (**Supplementary Figure S5**). Consequently, a contributing factor to the observed flesh discoloration in ‘M2’ pears may be attributed to the temperature induced diminished capacity for anthocyanin synthesis in the advanced developmental stages, compounded by the fruit’s enlargement, leading to a dilution effect on the pigment concentration.

This study enhances our understanding of the molecular mechanisms underlying flesh coloration in red pears and provides a foundation for further genetic improvement. Future studies should prioritize functional validation of key candidate transcription factors using genetic transformation and gene editing techniques such as CRISPR/Cas9 to clarify their precise roles and upstream regulatory networks. Further investigation is also warranted to elucidate the degradation pathways responsible for anthocyanin fading during late fruit maturation, as well as to explore how environmental factors like light and temperature interact with genetic components to regulate the distinctive annular pigmentation and its dynamic changes in the ‘M2’ cultivar. Concurrently, molecular markers should be developed to breed pear cultivars with stable and desirable flesh color. These research directions will be particularly valuable for developing new cultivars with stable and desirable color traits.

## Conclusions

In this study, we investigated the molecular and metabolic mechanisms driving the color conversion in pear flesh, focusing on the red-fleshed cultivar ‘M2’ and the white-fleshed cultivar ‘XM’. Through a comprehensive analysis of fruit development and metabolomic profiling, we identified anthocyanins, specifically cyanidin-3-O-galactoside, as the key metabolites responsible for the color differentiation in red-fleshed pear, with its concentration in ‘M2’ significantly exceeding that in ‘XM’ during the later stages of development. Transcriptomic analysis further revealed that anthocyanin biosynthesis in ‘M2’ is regulated by the upregulation of key structural genes, including *PcDFR*, *PcUFGT*, and *PcANS*, during fruit development. These genes are crucial for the synthesis of colored anthocyanidins, contributing to the red coloration in ‘M2’. Moreover, the identification of TFs such as *PcWER*, *PcbHLH062*, *PcGSTF12*, and *PcMYB114* highlighted their potential role in regulating the biosynthetic pathway of anthocyanins. Correlation analysis between these TFs and anthocyanin metabolites further reinforced their involvement in modulating red flesh pigmentation. These findings not only deepen our understanding of the molecular basis of pear flesh color development but also offer important implications for breeding strategies aimed at improving fruit quality.

## Data Availability

The RNA-Seq raw data from this study have been deposited in the National Center for Biotechnology Information (NCBI) Sequence Read Archive (SRA) under the accession number PRJNA1230845. https://www.ncbi.nlm.nih.gov/bioproject/PRJNA1230845/.
