# Supplementary material for: Integrated metabolomic and transcriptomic analyses elucidate anthocyanin-mediated flesh coloration mechanisms in red-fleshed pear
Source: Front Plant Sci. 2025 Oct 31;16:1670229. doi: 10.3389/fpls.2025.1670229 (PMC12615504; doi:10.3389/fpls.2025.1670229)
Supplement: Supplementary file 1 [file SupplementaryFile1.docx]

| **Table S1 Targeted metabolic assay compounds** | | | | | |
| --- | --- | --- | --- | --- | --- |
| **Index** | **Compounds** | **Group** | **MEAN** | **SD** | **SE** |
| Anthocyanidin_106 | Procyanidin B2 | XM | 69.52 | 7.69 | 4.44 |
| Anthocyanidin_108 | Procyanidin C1 | XM | 50.97 | 10.20 | 5.89 |
| Anthocyanidin_105 | Procyanidin B1 | XM | 11.54 | 1.14 | 0.66 |
| Anthocyanidin_42 | Rutin | XM | 1.64 | 0.32 | 0.18 |
| Anthocyanidin_41 | Quercetin-3-O-glucoside | XM | 1.49 | 0.29 | 0.17 |
| Anthocyanidin_40 | Naringenin-7-O-glucoside | XM | 1.04 | 0.17 | 0.10 |
| Anthocyanidin_107 | Procyanidin B3 | XM | 0.65 | 0.07 | 0.04 |
| Anthocyanidin_10 | Cyanidin-3-O-galactoside | XM | 0.42 | 0.02 | 0.01 |
| Anthocyanidin_38 | Kaempferol-3-O-rutinoside | XM | 0.31 | 0.07 | 0.04 |
| Anthocyanidin_23 | Delphinidin-3-O-(6-O-p-coumaroyl)-glucoside | XM | 0.18 | 0.05 | 0.03 |
| Anthocyanidin_94 | Petunidin-3-O-(6-O-p-coumaroyl)-glucoside | XM | 0.18 | 0.05 | 0.03 |
| Anthocyanidin_37 | Dihydromyricetin | XM | 0.10 | 0.01 | 0.01 |
| Anthocyanidin_85 | Peonidin-3-O-galactoside | XM | 0.07 | 0.01 | 0.00 |
| Anthocyanidin_78 | Peonidin-3,5-O-diglucoside | XM | 0.03 | 0.00 | 0.00 |
| Anthocyanidin_104 | Procyanidin A2 | XM | 0.03 | 0.00 | 0.00 |
| Anthocyanidin_06 | Cyanidin-3-O-(6-O-p-coumaroyl)-glucoside | XM | 0.02 | 0.00 | 0.00 |
| Anthocyanidin_04 | Cyanidin-3,5-O-diglucoside | XM | 0.02 | 0.00 | 0.00 |
| Anthocyanidin_39 | Naringenin | XM | 0.02 | 0.00 | 0.00 |
| Anthocyanidin_21 | Delphinidin-3-O-(6-O-malonyl-beta-D-glucoside) | XM | 0.02 | 0.00 | 0.00 |
| Anthocyanidin_11 | Cyanidin-3-O-glucoside | XM | 0.01 | 0.00 | 0.00 |
| Anthocyanidin_29 | Delphinidin-3-O-rutinoside | XM | 0.01 | 0.00 | 0.00 |
| Anthocyanidin_16 | Cyanidin-3-O-xyloside | XM | 0.00 | 0.00 | 0.00 |
| Anthocyanidin_35 | Chalcone | XM | 0.00 | 0.00 | 0.00 |
| Anthocyanidin_27 | Delphinidin-3-O-glucoside | XM | 0.00 | 0.00 | 0.00 |
| Anthocyanidin_14 | Cyanidin-3-O-sambubioside | XM | 0.00 | 0.00 | 0.00 |
| Anthocyanidin_08 | Cyanidin-3-O-arabinoside | XM | 0.00 | 0.00 | 0.00 |
| Anthocyanidin_05 | Cyanidin-3-O-(6-O-malonyl-beta-D-glucoside) | XM | 0.00 | 0.00 | 0.00 |
| Anthocyanidin_12 | Cyanidin-3-O-rutinoside | XM | 0.00 | 0.00 | 0.00 |
| Anthocyanidin_15 | Cyanidin-3-O-sophoroside | XM | 0.00 | 0.00 | 0.00 |
| Anthocyanidin_25 | Delphinidin-3-O-arabinoside | XM | 0.00 | 0.00 | 0.00 |
| Anthocyanidin_51 | Malvidin-3-O-glucoside | XM | 0.00 | 0.00 | 0.00 |
| Anthocyanidin_56 | Pelargonidin | XM | 0.00 | 0.00 | 0.00 |
| Anthocyanidin_68 | Pelargonidin-3-O-glucoside | XM | 0.00 | 0.00 | 0.00 |
| Anthocyanidin_80 | Peonidin-3-O-(6-O-p-coumaroyl)-glucoside | XM | 0.00 | 0.00 | 0.00 |
| Anthocyanidin_82 | Peonidin-3-O-arabinoside | XM | 0.00 | 0.00 | 0.00 |
| Anthocyanidin_86 | Peonidin-3-O-glucoside | XM | 0.00 | 0.00 | 0.00 |
| Anthocyanidin_10 | Cyanidin-3-O-galactoside | M2 | 497.78 | 63.87 | 36.88 |
| Anthocyanidin_106 | Procyanidin B2 | M2 | 116.21 | 15.40 | 8.89 |
| Anthocyanidin_108 | Procyanidin C1 | M2 | 76.31 | 10.82 | 6.25 |
| Anthocyanidin_105 | Procyanidin B1 | M2 | 46.74 | 6.41 | 3.70 |
| Anthocyanidin_41 | Quercetin-3-O-glucoside | M2 | 17.40 | 3.14 | 1.81 |
| Anthocyanidin_11 | Cyanidin-3-O-glucoside | M2 | 10.92 | 1.19 | 0.69 |
| Anthocyanidin_42 | Rutin | M2 | 8.16 | 2.77 | 1.60 |
| Anthocyanidin_107 | Procyanidin B3 | M2 | 2.75 | 0.39 | 0.23 |
| Anthocyanidin_08 | Cyanidin-3-O-arabinoside | M2 | 2.19 | 0.29 | 0.16 |
| Anthocyanidin_40 | Naringenin-7-O-glucoside | M2 | 0.77 | 0.11 | 0.06 |
| Anthocyanidin_85 | Peonidin-3-O-galactoside | M2 | 0.68 | 0.06 | 0.04 |
| Anthocyanidin_38 | Kaempferol-3-O-rutinoside | M2 | 0.63 | 0.20 | 0.12 |
| Anthocyanidin_23 | Delphinidin-3-O-(6-O-p-coumaroyl)-glucoside | M2 | 0.46 | 0.16 | 0.09 |
| Anthocyanidin_94 | Petunidin-3-O-(6-O-p-coumaroyl)-glucoside | M2 | 0.41 | 0.05 | 0.03 |
| Anthocyanidin_04 | Cyanidin-3,5-O-diglucoside | M2 | 0.10 | 0.01 | 0.00 |
| Anthocyanidin_37 | Dihydromyricetin | M2 | 0.09 | 0.01 | 0.01 |
| Anthocyanidin_78 | Peonidin-3,5-O-diglucoside | M2 | 0.05 | 0.01 | 0.00 |
| Anthocyanidin_06 | Cyanidin-3-O-(6-O-p-coumaroyl)-glucoside | M2 | 0.05 | 0.01 | 0.00 |
| Anthocyanidin_39 | Naringenin | M2 | 0.02 | 0.00 | 0.00 |
| Anthocyanidin_15 | Cyanidin-3-O-sophoroside | M2 | 0.02 | 0.00 | 0.00 |
| Anthocyanidin_12 | Cyanidin-3-O-rutinoside | M2 | 0.02 | 0.00 | 0.00 |
| Anthocyanidin_27 | Delphinidin-3-O-glucoside | M2 | 0.02 | 0.00 | 0.00 |
| Anthocyanidin_68 | Pelargonidin-3-O-glucoside | M2 | 0.01 | 0.00 | 0.00 |
| Anthocyanidin_56 | Pelargonidin | M2 | 0.01 | 0.00 | 0.00 |
| Anthocyanidin_21 | Delphinidin-3-O-(6-O-malonyl-beta-D-glucoside) | M2 | 0.01 | 0.00 | 0.00 |
| Anthocyanidin_80 | Peonidin-3-O-(6-O-p-coumaroyl)-glucoside | M2 | 0.01 | 0.00 | 0.00 |
| Anthocyanidin_25 | Delphinidin-3-O-arabinoside | M2 | 0.01 | 0.00 | 0.00 |
| Anthocyanidin_14 | Cyanidin-3-O-sambubioside | M2 | 0.01 | 0.00 | 0.00 |
| Anthocyanidin_29 | Delphinidin-3-O-rutinoside | M2 | 0.01 | 0.00 | 0.00 |
| Anthocyanidin_86 | Peonidin-3-O-glucoside | M2 | 0.00 | 0.00 | 0.00 |
| Anthocyanidin_16 | Cyanidin-3-O-xyloside | M2 | 0.00 | 0.00 | 0.00 |
| Anthocyanidin_35 | Chalcone | M2 | 0.00 | 0.00 | 0.00 |
| Anthocyanidin_51 | Malvidin-3-O-glucoside | M2 | 0.00 | 0.00 | 0.00 |
| Anthocyanidin_05 | Cyanidin-3-O-(6-O-malonyl-beta-D-glucoside) | M2 | 0.00 | 0.00 | 0.00 |
| Anthocyanidin_82 | Peonidin-3-O-arabinoside | M2 | 0.00 | 0.00 | 0.00 |
| Anthocyanidin_104 | Procyanidin A2 | M2 | 0.00 | 0.00 | 0.00 |
| Carotenoid_59 | lutein | XM | 35.22 | 9.90 | 5.72 |
| Carotenoid_58 | neoxanthin | XM | 2.78 | 0.55 | 0.32 |
| Carotenoid_04 | β-carotene | XM | 2.52 | 0.50 | 0.29 |
| Carotenoid_57 | violaxanthin | XM | 1.31 | 0.22 | 0.13 |
| Carotenoid_15 | lutein dilaurate | XM | 1.15 | 0.22 | 0.13 |
| Carotenoid_37 | violaxanthin dipalmitate | XM | 0.84 | 0.29 | 0.17 |
| Carotenoid_12 | lutein palmitate | XM | 0.54 | 0.10 | 0.06 |
| Carotenoid_56 | zeaxanthin | XM | 0.40 | 0.08 | 0.05 |
| Carotenoid_60 | β-cryptoxanthin | XM | 0.28 | 0.06 | 0.03 |
| Carotenoid_18 | lutein dipalmitate | XM | 0.24 | 0.04 | 0.02 |
| Carotenoid_17 | lutein dimyristate | XM | 0.13 | 0.03 | 0.02 |
| Carotenoid_01 | α-carotene | XM | 0.11 | 0.01 | 0.01 |
| Carotenoid_29 | violaxanthin myristate | XM | 0.06 | 0.01 | 0.01 |
| Carotenoid_51 | β-cryptoxanthin laurate | XM | 0.05 | 0.01 | 0.01 |
| Carotenoid_10 | lutein laurate | XM | 0.04 | 0.01 | 0.00 |
| Carotenoid_64 | α-cryptoxanthin | XM | 0.03 | 0.00 | 0.00 |
| Carotenoid_14 | 5,6epoxy-luttein dilaurate | XM | 0.02 | 0.00 | 0.00 |
| Carotenoid_28 | violaxanthin laurate | XM | 0.01 | 0.00 | 0.00 |
| Carotenoid_27 | violaxanthin dibutyrate | XM | 0.01 | 0.01 | 0.00 |
| Carotenoid_09 | lutein caprate | XM | 0.01 | 0.00 | 0.00 |
| Carotenoid_59 | lutein | M2 | 22.17 | 4.35 | 2.51 |
| Carotenoid_58 | neoxanthin | M2 | 1.34 | 0.12 | 0.07 |
| Carotenoid_04 | β-carotene | M2 | 1.21 | 0.19 | 0.11 |
| Carotenoid_37 | violaxanthin dipalmitate | M2 | 1.00 | 0.31 | 0.18 |
| Carotenoid_57 | violaxanthin | M2 | 0.80 | 0.13 | 0.07 |
| Carotenoid_15 | lutein dilaurate | M2 | 0.43 | 0.05 | 0.03 |
| Carotenoid_56 | zeaxanthin | M2 | 0.35 | 0.05 | 0.03 |
| Carotenoid_12 | lutein palmitate | M2 | 0.17 | 0.01 | 0.00 |
| Carotenoid_18 | lutein dipalmitate | M2 | 0.15 | 0.01 | 0.01 |
| Carotenoid_60 | β-cryptoxanthin | M2 | 0.12 | 0.01 | 0.01 |
| Carotenoid_01 | α-carotene | M2 | 0.07 | 0.01 | 0.01 |
| Carotenoid_17 | lutein dimyristate | M2 | 0.05 | 0.01 | 0.00 |
| Carotenoid_27 | violaxanthin dibutyrate | M2 | 0.02 | 0.03 | 0.02 |
| Carotenoid_51 | β-cryptoxanthin laurate | M2 | 0.01 | 0.00 | 0.00 |
| Carotenoid_09 | lutein caprate | M2 | 0.00 | 0.00 | 0.00 |
| Carotenoid_10 | lutein laurate | M2 | 0.00 | 0.00 | 0.00 |
| Carotenoid_14 | 5,6epoxy-luttein dilaurate | M2 | 0.00 | 0.00 | 0.00 |
| Carotenoid_28 | violaxanthin laurate | M2 | 0.00 | 0.00 | 0.00 |
| Carotenoid_29 | violaxanthin myristate | M2 | 0.00 | 0.00 | 0.00 |
| Carotenoid_64 | α-cryptoxanthin | M2 | 0.00 | 0.00 | 0.00 |

| **Table S2 Sequencing output statistics table** | | | | | | | |
| --- | --- | --- | --- | --- | --- | --- | --- |
| **Sample** | **Raw Reads** | **Clean Reads** | **Clean Base(G)** | **Error Rate(%)** | **Q20(%)** | **Q30(%)** | **GC Content(%)** |
| M2-1 | 41644166 | 40815974 | 6.12 | 0.02 | 98.25 | 94.75 | 47.15 |
| M2-2 | 41812094 | 41077620 | 6.16 | 0.02 | 98.24 | 94.71 | 47.23 |
| M2-3 | 57982974 | 56996324 | 8.55 | 0.02 | 98.31 | 94.98 | 47.13 |
| XM-1 | 43168380 | 42218776 | 6.33 | 0.02 | 98.31 | 94.86 | 47.39 |
| XM-2 | 50681178 | 49762828 | 7.46 | 0.03 | 98 | 94.15 | 47.39 |
| XM-3 | 42774860 | 41947958 | 6.29 | 0.02 | 98.11 | 94.36 | 47.3 |

| **Table S3 Comparison Statistics Table** | | | | | | | | |
| --- | --- | --- | --- | --- | --- | --- | --- | --- |
| **Sample** | **Total Reads** | **Reads mapped** | **Unique mapped** | **Multi mapped** | **Read1 mapped** | **Read2 mapped** | **'+' mapped** | **'-' mapped** |
| M2-1 | 40815974 | 37885400(92.82%) | 36976992(90.59%) | 908408(2.23%) | 18513466(45.36%) | 18463526(45.24%) | 18496156(45.32%) | 18480836(45.28%) |
| M2-2 | 41077620 | 38149241(92.87%) | 37257347(90.70%) | 891894(2.17%) | 18655117(45.41%) | 18602230(45.29%) | 18640888(45.38%) | 18616459(45.32%) |
| M2-3 | 56996324 | 52854071(92.73%) | 51542018(90.43%) | 1312053(2.30%) | 25789120(45.25%) | 25752898(45.18%) | 25784772(45.24%) | 25757246(45.19%) |
| XM-1 | 42218776 | 39598775(93.79%) | 38651566(91.55%) | 947209(2.24%) | 19349348(45.83%) | 19302218(45.72%) | 19333273(45.79%) | 19318293(45.76%) |
| XM-2 | 49762828 | 46378570(93.20%) | 45258460(90.95%) | 1120110(2.25%) | 22653880(45.52%) | 22604580(45.42%) | 22633539(45.48%) | 22624921(45.47%) |
| XM-3 | 41947958 | 39275650(93.63%) | 38336305(91.39%) | 939345(2.24%) | 19207934(45.79%) | 19128371(45.60%) | 19173523(45.71%) | 19162782(45.68%) |

| **Table S4 Mobile phase gradient elution program** | | |
| --- | --- | --- |
| **Time(min)** | **Mobile phase A** | **Mobile phase B** |
| 0 | 100 | 0 |
| 30 | 75 | 25 |
| 45 | 60 | 40 |
| 60 | 40 | 60 |
| 61 | 100 | 0 |
| 80 | 100 | 0 |

| **Table S5 Correlation between TF and Anthocyanins** | | | | | | | | | | |
| --- | --- | --- | --- | --- | --- | --- | --- | --- | --- | --- |
| **geneID** | **symbol** | **Cyanidin-3-O-galactoside** | **Procyanidin B2** | **Procyanidin C1** | **Procyanidin B1** | **Quercetin-3-O-glucoside** | **Cyanidin-3-O-glucoside** | **Rutin** | **Procyanidin B3** | **Cyanidin-3-O-arabinoside** |
| pycom09g10130 | WER | 0.92 | 0.00 | 0.00 | 0.89 | 0.88 | 0.92 | 0.94 | 0.89 | 0.91 |
| pycom16g19130 | MYB2 | 0.99 | 0.96 | 0.88 | 0.99 | 0.98 | 0.99 | 0.84 | 0.99 | 0.99 |
| pycom02g07060 | MYB12 | 0.10 | 0.10 | 0.10 | 0.10 | 0.10 | 0.10 | 0.00 | 0.10 | 0.10 |
| pycom15g24200 | HHO5 | 0.95 | 0.85 | 0.00 | 0.93 | 0.95 | 0.96 | 0.88 | 0.93 | 0.95 |
| pycom13g07260 | KUA1 | 0.90 | 0.00 | 0.00 | 0.88 | 0.94 | 0.91 | 0.00 | 0.88 | 0.90 |
| pycom06g19360 | MYB62 | 0.00 | 0.00 | 0.00 | 0.00 | 0.00 | 0.00 | 0.00 | 0.00 | 0.00 |
| pycom06g17130 | MYB306 | 0.00 | 0.00 | 0.00 | 0.00 | 0.00 | 0.00 | 0.00 | 0.00 | 0.00 |
| pycom16g07230 | KUA1 | 0.98 | 0.95 | 0.85 | 0.97 | 0.93 | 0.98 | 0.94 | 0.97 | 0.97 |
| pycom09g08660 | PHL6 | 0.00 | 0.10 | 0.10 | 0.00 | 0.10 | 0.00 | 0.00 | 0.00 | 0.00 |
| pycom12g10860 | MYB27 | 0.00 | 0.00 | 0.00 | 0.00 | 0.00 | 0.00 | 0.00 | 0.00 | 0.00 |
| pycom11g27640 | MYB11 | 0.00 | 0.00 | 0.00 | 0.00 | 0.00 | 0.00 | 0.00 | 0.00 | 0.00 |
| pycom12g18500 | MYB61 | 0.94 | 0.85 | 0.00 | 0.91 | 0.89 | 0.94 | 0.94 | 0.91 | 0.93 |
| pycom05g25770 | MYB114 | 0.99 | 0.93 | 0.85 | 0.99 | 0.98 | 1.00 | 0.89 | 0.98 | 0.99 |
| pycom04g19460 | At5g28040 | 0.00 | 0.00 | 0.00 | 0.00 | 0.00 | 0.00 | 0.00 | 0.00 | 0.00 |
| pycom11g27650 | -- | 0.00 | 0.00 | 0.00 | 0.00 | 0.00 | 0.00 | 0.00 | 0.00 | 0.00 |
| pycom15g26690 | -- | 0.92 | 0.00 | 0.00 | 0.88 | 0.90 | 0.92 | 0.90 | 0.88 | 0.91 |
| pycom14g15700 | KUA1 | 0.10 | 0.10 | 0.00 | 0.10 | 0.10 | 0.10 | 0.10 | 0.10 | 0.10 |
| pycom03g23560 | MYB12 | 0.00 | 0.00 | 0.00 | 0.00 | 0.00 | 0.00 | 0.00 | 0.00 | 0.00 |
| pycom04g16420 | -- | 0.10 | 0.00 | 0.00 | 0.10 | 0.00 | 0.10 | 0.10 | 0.10 | 0.10 |
| pycom16g25380 | PHL11 | 0.93 | 0.83 | 0.00 | 0.91 | 0.91 | 0.93 | 0.92 | 0.90 | 0.93 |
| pycom10g25800 | MYB4 | 0.00 | 0.00 | 0.00 | 0.00 | 0.00 | 0.00 | 0.00 | 0.00 | 0.00 |
| pycom06g11120 | bHLH28 | 0.00 | 0.00 | 0.00 | 0.00 | 0.00 | 0.00 | 0.10 | 0.00 | 0.00 |
| pycom12g08210 | bHLH106 | 0.10 | 0.10 | 0.10 | 0.10 | 0.10 | 0.10 | 0.10 | 0.10 | 0.10 |
| pycom13g18420 | bHLH104 | 0.00 | 0.00 | 0.00 | 0.00 | 0.00 | 0.00 | 0.00 | 0.00 | 0.00 |
| pycom15g35280 | bHLH93 | 0.00 | 0.00 | 0.00 | 0.00 | 0.00 | 0.00 | 0.00 | 0.00 | 0.00 |
| pycom05g18670 | bHLH155 | 0.89 | 0.00 | 0.00 | 0.86 | 0.92 | 0.90 | 0.00 | 0.86 | 0.89 |
| pycom08g17980 | bHLH93 | 0.10 | 0.10 | 0.10 | 0.10 | 0.10 | 0.10 | 0.00 | 0.10 | 0.10 |
| pycom04g04320 | bHLH153 | 0.10 | 0.10 | 0.10 | 0.10 | 0.10 | 0.10 | 0.10 | 0.10 | 0.10 |
| pycom04g17000 | bHLH149 | 0.00 | 0.00 | 0.00 | 0.00 | 0.00 | 0.00 | 0.00 | 0.00 | 0.00 |
| pycom02g26450 | bHLH130 | 0.95 | 0.83 | 0.00 | 0.93 | 0.95 | 0.95 | 0.86 | 0.92 | 0.95 |
| pycom04g02000 | bHLH62 | 0.00 | 0.00 | 0.00 | 0.00 | 0.00 | 0.00 | 0.00 | 0.00 | 0.00 |
| pycom11g25430 | bHLH | 0.94 | 0.83 | 0.00 | 0.91 | 0.87 | 0.93 | 0.88 | 0.91 | 0.93 |
| pycom12g05580 | bHLH60 | 0.95 | 0.85 | 0.00 | 0.93 | 0.94 | 0.96 | 0.90 | 0.93 | 0.95 |
| pycom16g23100 | IBL1 | 0.10 | 0.10 | 0.10 | 0.10 | 0.10 | 0.10 | 0.10 | 0.10 | 0.10 |
| pycom07g20270 | bHLH51 | 0.00 | 0.00 | 0.00 | 0.00 | 0.00 | 0.00 | 0.00 | 0.00 | 0.00 |
| pycom06g16970 | bHLH062 | 0.96 | 0.88 | 0.00 | 0.94 | 0.89 | 0.95 | 0.98 | 0.94 | 0.95 |
| pycom07g23850 | bHLH120 | 0.00 | 0.00 | 0.00 | 0.00 | 0.00 | 0.00 | 0.00 | 0.00 | 0.00 |
| pycom10g16370 | bHLH3 | 0.00 | 0.00 | 0.00 | 0.00 | 0.00 | 0.00 | 0.00 | 0.00 | 0.00 |
| pycom14g15170 | bHLH162 | 0.00 | 0.00 | 0.00 | 0.00 | 0.00 | 0.00 | 0.00 | 0.00 | 0.00 |
| pycom01g11720 | -- | 0.10 | 0.00 | 0.00 | 0.10 | 0.10 | 0.10 | 0.00 | 0.10 | 0.10 |
| pycom06g17320 | NAC100 | 0.00 | 0.00 | 0.00 | 0.00 | 0.00 | 0.00 | 0.00 | 0.00 | 0.00 |
| pycom07g15840 | NAC078 | 0.10 | 0.10 | 0.00 | 0.10 | 0.10 | 0.10 | 0.10 | 0.10 | 0.10 |
| pycom07g15860 | -- | 0.99 | 0.97 | 0.90 | 1.00 | 0.97 | 0.99 | 0.89 | 1.00 | 0.99 |
| pycom07g15870 | NAC014 | 0.00 | 0.00 | 0.00 | 0.00 | 0.00 | 0.00 | 0.00 | 0.00 | 0.00 |
| pycom07g15890 | NAC014 | 0.00 | 0.00 | 0.00 | 0.00 | 0.00 | 0.00 | 0.00 | 0.00 | 0.00 |
| pycom13g05640 | NAC029 | 0.10 | 0.10 | 0.10 | 0.10 | 0.10 | 0.10 | 0.10 | 0.10 | 0.10 |
| pycom15g12330 | NAC081 | 0.83 | 0.00 | 0.00 | 0.00 | 0.00 | 0.83 | 0.85 | 0.00 | 0.83 |
| pycom16g04220 | SOG1 | 0.10 | 0.10 | 0.10 | 0.10 | 0.10 | 0.10 | 0.10 | 0.10 | 0.10 |
| pycom17g04870 | NAC100 | 0.00 | 0.00 | 0.00 | 0.00 | 0.00 | 0.00 | 0.00 | 0.00 | 0.00 |
| pycom01g11680 | NAC014 | 0.00 | 0.00 | 0.00 | 0.00 | 0.00 | 0.00 | 0.00 | 0.00 | 0.00 |
| pycom06g04360 | ERF5 | 0.10 | 0.00 | 0.10 | 0.10 | 0.10 | 0.10 | 0.00 | 0.10 | 0.10 |
| pycom14g12170 | ERF114 | 0.00 | 0.00 | 0.00 | 0.00 | 0.00 | 0.00 | 0.00 | 0.00 | 0.00 |
| pycom01g20680 | ERF025 | 0.00 | 0.87 | 0.87 | 0.00 | 0.00 | 0.00 | 0.00 | 0.00 | 0.00 |
| pycom09g16830 | ERF053 | 0.99 | 0.98 | 0.90 | 0.99 | 0.95 | 0.99 | 0.89 | 0.99 | 0.99 |
| pycom07g08160 | TINY | 0.97 | 0.92 | 0.00 | 0.96 | 0.90 | 0.96 | 0.91 | 0.96 | 0.96 |
| pycom16g11140 | ERF114 | 0.00 | 0.00 | 0.00 | 0.00 | 0.00 | 0.00 | 0.00 | 0.00 | 0.00 |
| pycom16g03760 | ERF118 | 0.10 | 0.10 | 0.10 | 0.10 | 0.10 | 0.10 | 0.10 | 0.10 | 0.10 |
| pycom08g05000 | ERF003 | 0.10 | 0.10 | 0.00 | 0.10 | 0.10 | 0.10 | 0.10 | 0.10 | 0.10 |
| pycom12g06470 | At2g41710 | 0.95 | 0.86 | 0.00 | 0.93 | 0.92 | 0.95 | 0.93 | 0.93 | 0.95 |
| pycom01g22600 | WRKY53 | 0.00 | 0.00 | 0.00 | 0.00 | 0.00 | 0.00 | 0.00 | 0.00 | 0.00 |
| pycom02g14340 | WRKY7 | 0.97 | 0.90 | 0.00 | 0.95 | 0.92 | 0.97 | 0.96 | 0.95 | 0.96 |
| pycom04g15490 | WRKY38 | 0.00 | 0.00 | 0.00 | 0.00 | 0.00 | 0.00 | 0.00 | 0.00 | 0.00 |
| pycom05g26710 | WRKY28 | 0.10 | 0.10 | 0.10 | 0.10 | 0.10 | 0.10 | 0.10 | 0.10 | 0.10 |
| pycom07g14630 | WRKY30 | 0.10 | 0.10 | 0.00 | 0.10 | 0.10 | 0.10 | 0.10 | 0.10 | 0.10 |
| pycom08g07690 | WRKY7 | 0.00 | 0.00 | 0.00 | 0.00 | 0.00 | 0.00 | 0.00 | 0.00 | 0.00 |
| pycom09g04600 | WRKY1 | 0.10 | 0.10 | 0.10 | 0.10 | 0.10 | 0.10 | 0.00 | 0.10 | 0.10 |
| pycom09g07040 | WRKY71 | 0.91 | 0.88 | 0.00 | 0.90 | 0.00 | 0.90 | 0.95 | 0.91 | 0.90 |
| pycom09g19270 | -- | 0.10 | 0.10 | 0.10 | 0.10 | 0.10 | 0.10 | 0.10 | 0.10 | 0.10 |
| pycom13g10630 | WRKY75 | 0.88 | 0.93 | 0.83 | 0.89 | 0.00 | 0.86 | 0.00 | 0.89 | 0.87 |
| pycom13g21150 | WRKY11 | 0.98 | 0.98 | 0.91 | 0.98 | 0.94 | 0.97 | 0.91 | 0.98 | 0.98 |
| pycom15g09730 | WRKY11 | 0.10 | 0.00 | 0.00 | 0.10 | 0.10 | 0.10 | 0.00 | 0.10 | 0.10 |
| pycom15g25080 | WRKY7 | 0.10 | 0.10 | 0.10 | 0.10 | 0.10 | 0.10 | 0.00 | 0.10 | 0.10 |
| pycom16g05540 | WRKY57 | 0.89 | 0.00 | 0.00 | 0.86 | 0.90 | 0.90 | 0.81 | 0.86 | 0.89 |
| pycom16g12920 | WRKY48 | 0.00 | 0.10 | 0.10 | 0.00 | 0.00 | 0.00 | 0.00 | 0.00 | 0.00 |
| pycom16g20580 | WRKY11 | 0.00 | 0.00 | 0.00 | 0.00 | 0.00 | 0.00 | 0.89 | 0.00 | 0.00 |
| pycom17g22700 | WRKY40 | 0.00 | 0.00 | 0.00 | 0.00 | 0.00 | 0.00 | 0.00 | 0.00 | 0.00 |
| pycom02g16120 | AGL6 | 0.00 | 0.00 | 0.00 | 0.00 | 0.82 | 0.00 | 0.00 | 0.00 | 0.00 |
| pycom02g25320 | AGL15 | 0.88 | 0.00 | 0.00 | 0.85 | 0.84 | 0.88 | 0.92 | 0.84 | 0.87 |
| pycom13g10590 | FBP2 | 0.00 | 0.00 | 0.00 | 0.00 | 0.00 | 0.00 | 0.87 | 0.00 | 0.00 |

| **Table S6 FPKM of TFs** | | | | | | | | |
| --- | --- | --- | --- | --- | --- | --- | --- | --- |
| **geneID** | **symbol** | **XM-1_fpkm** | **XM-2_fpkm** | **XM-3_fpkm** | **M2-1_fpkm** | **M2-2_fpkm** | **M2-3_fpkm** | **log2FoldChange** |
| pycom09g10130 | WER | 27.43 | 25.72 | 31.96 | 15.30 | 15.89 | 16.57 | 0.85 |
| pycom16g19130 | MYB2 | 1.23 | 1.22 | 0.88 | 0.08 | 0.13 | 0.00 | #N/A |
| pycom02g07060 | MYB12 | 6.06 | 4.81 | 5.26 | 8.54 | 7.85 | 8.74 | -0.63 |
| pycom15g24200 | HHO5 | 7.19 | 7.70 | 8.34 | 3.57 | 3.80 | 3.46 | 1.11 |
| pycom13g07260 | KUA1 | 8.25 | 10.47 | 9.93 | 5.78 | 3.88 | 5.01 | 0.98 |
| pycom06g19360 | MYB62 | #N/A | #N/A | #N/A | #N/A | #N/A | #N/A | #N/A |
| pycom06g17130 | MYB306 | 0.62 | 0.67 | 0.52 | 0.50 | 0.76 | 1.25 | #N/A |
| pycom16g07230 | KUA1 | 19.69 | 17.19 | 17.99 | 8.47 | 10.92 | 9.29 | 0.94 |
| pycom09g08660 | PHL6 | 11.19 | 9.80 | 11.88 | 12.71 | 12.02 | 12.50 | -0.17 |
| pycom12g10860 | MYB27 | 0.58 | 0.33 | 1.19 | 0.40 | 0.41 | 0.72 | #N/A |
| pycom11g27640 | MYB11 | 0.05 | 0.00 | 0.00 | 0.00 | 0.04 | 0.10 | #N/A |
| pycom12g18500 | MYB61 | 31.16 | 28.81 | 33.21 | 18.99 | 17.74 | 21.49 | 0.69 |
| pycom05g25770 | MYB114 | 47.32 | 47.82 | 44.45 | 0.00 | 0.00 | 0.00 | 11.73 |
| pycom04g19460 | At5g28040 | 5.88 | 5.67 | 5.78 | 5.17 | 5.43 | 5.73 | 0.09 |
| pycom11g27650 | -- | #N/A | #N/A | #N/A | #N/A | #N/A | #N/A | #N/A |
| pycom15g26690 | -- | 51.50 | 51.72 | 56.52 | 42.14 | 40.03 | 41.59 | 0.38 |
| pycom14g15700 | KUA1 | 210.97 | 208.18 | 195.28 | 292.66 | 301.97 | 293.89 | -0.52 |
| pycom03g23560 | MYB12 | 0.12 | 0.08 | 0.23 | 0.23 | 0.30 | 0.16 | #N/A |
| pycom04g16420 | -- | 8.84 | 11.96 | 9.80 | 14.32 | 14.91 | 18.44 | -0.63 |
| pycom16g25380 | PHL11 | 4.77 | 4.65 | 5.97 | 1.24 | 1.55 | 1.90 | 1.73 |
| pycom10g25800 | MYB4 | 0.25 | 0.57 | 0.06 | 0.55 | 0.67 | 0.16 | #N/A |
| pycom06g11120 | bHLH28 | 13.48 | 14.67 | 12.78 | 15.18 | 16.33 | 15.22 | -0.19 |
| pycom12g08210 | bHLH106 | 3.40 | 3.61 | 3.14 | 8.05 | 5.86 | 6.09 | -0.97 |
| pycom13g18420 | bHLH104 | 53.79 | 50.89 | 49.25 | 49.45 | 48.50 | 50.83 | 0.06 |
| pycom15g35280 | bHLH93 | 0.15 | 0.44 | 0.28 | 0.42 | 0.44 | 0.14 | #N/A |
| pycom05g18670 | bHLH155 | 51.72 | 53.80 | 54.67 | 48.45 | 48.24 | 47.25 | 0.16 |
| pycom08g17980 | bHLH93 | 5.59 | 3.49 | 6.16 | 11.86 | 11.08 | 11.30 | -1.15 |
| pycom04g04320 | bHLH153 | 47.39 | 61.38 | 59.19 | 93.36 | 84.98 | 93.51 | -0.68 |
| pycom04g17000 | bHLH149 | 24.00 | 18.93 | 14.88 | 19.25 | 21.71 | 21.32 | -0.11 |
| pycom02g26450 | bHLH130 | 7.46 | 8.07 | 8.58 | 4.16 | 3.05 | 3.53 | 1.18 |
| pycom04g02000 | bHLH62 | 39.58 | 36.03 | 36.23 | 36.39 | 35.53 | 37.23 | 0.04 |
| pycom11g25430 | bHLH | 6.41 | 6.04 | 6.13 | 5.54 | 5.00 | 5.00 | 0.27 |
| pycom12g05580 | bHLH60 | 38.99 | 39.30 | 43.10 | 24.09 | 21.66 | 23.83 | 0.82 |
| pycom16g23100 | IBL1 | 0.00 | 0.00 | 0.25 | 2.36 | 1.44 | 1.70 | #N/A |
| pycom07g20270 | bHLH51 | 3.25 | 3.21 | 4.14 | 4.13 | 3.87 | 4.59 | -0.23 |
| pycom06g16970 | bHLH062 | 48.94 | 40.95 | 48.38 | 27.03 | 25.97 | 26.43 | 0.81 |
| pycom07g23850 | bHLH120 | #N/A | #N/A | #N/A | #N/A | #N/A | #N/A | #N/A |
| pycom10g16370 | bHLH3 | 22.48 | 24.82 | 25.61 | 22.05 | 26.43 | 24.23 | 0.01 |
| pycom14g15170 | bHLH162 | #N/A | #N/A | #N/A | #N/A | #N/A | #N/A | #N/A |
| pycom01g11720 | -- | 2.77 | 2.67 | 2.82 | 4.12 | 4.97 | 6.33 | -0.89 |
| pycom06g17320 | NAC100 | 6.42 | 6.46 | 7.11 | 6.55 | 5.75 | 6.34 | 0.12 |
| pycom07g15840 | NAC078 | 4.07 | 3.54 | 3.64 | 6.19 | 6.92 | 6.07 | -0.77 |
| pycom07g15860 | -- | 31.52 | 29.68 | 27.12 | 10.70 | 13.80 | 13.14 | 1.23 |
| pycom07g15870 | NAC014 | 6.41 | 5.49 | 6.85 | 6.95 | 7.91 | 6.22 | -0.16 |
| pycom07g15890 | NAC014 | 18.68 | 15.73 | 17.67 | 15.08 | 15.66 | 17.60 | 0.12 |
| pycom13g05640 | NAC029 | 11.90 | 15.42 | 15.68 | 20.52 | 19.27 | 18.23 | -0.42 |
| pycom15g12330 | NAC081 | 4.94 | 4.76 | 5.39 | 4.41 | 3.65 | 3.83 | 0.36 |
| pycom16g04220 | SOG1 | 21.75 | 20.84 | 21.53 | 28.64 | 28.15 | 28.23 | -0.40 |
| pycom17g04870 | NAC100 | 0.39 | 0.33 | 0.60 | 0.21 | 0.18 | 0.43 | #N/A |
| pycom01g11680 | NAC014 | 58.10 | 87.07 | 81.82 | 73.48 | 65.79 | 65.16 | 0.16 |
| pycom06g04360 | ERF5 | 0.07 | 0.00 | 0.00 | 0.19 | 0.11 | 0.13 | #N/A |
| pycom14g12170 | ERF114 | #N/A | #N/A | #N/A | #N/A | #N/A | #N/A | #N/A |
| pycom01g20680 | ERF025 | 0.80 | 0.63 | 0.17 | 0.00 | 0.00 | 0.38 | #N/A |
| pycom09g16830 | ERF053 | 2.42 | 2.13 | 1.86 | 0.38 | 0.80 | 0.56 | 1.86 |
| pycom07g08160 | TINY | 7.05 | 6.38 | 6.43 | 5.01 | 5.20 | 4.58 | 0.43 |
| pycom16g11140 | ERF114 | 0.07 | 0.00 | 0.15 | 0.07 | 0.17 | 0.20 | #N/A |
| pycom16g03760 | ERF118 | 8.87 | 9.52 | 9.59 | 17.38 | 16.00 | 17.59 | -0.85 |
| pycom08g05000 | ERF003 | 7.87 | 7.90 | 5.97 | 18.60 | 20.80 | 19.92 | -1.45 |
| pycom12g06470 | At2g41710 | 131.12 | 127.31 | 141.22 | 86.45 | 84.60 | 91.97 | 0.61 |
| pycom01g22600 | WRKY53 | #N/A | #N/A | #N/A | #N/A | #N/A | #N/A | #N/A |
| pycom02g14340 | WRKY7 | 28.14 | 25.46 | 28.37 | 16.32 | 17.49 | 17.44 | 0.69 |
| pycom04g15490 | WRKY38 | 0.00 | 0.06 | 0.00 | 0.05 | 0.14 | 0.00 | #N/A |
| pycom05g26710 | WRKY28 | 4.47 | 4.06 | 4.55 | 12.16 | 11.41 | 11.73 | -1.42 |
| pycom07g14630 | WRKY30 | 0.64 | 0.38 | 0.18 | 4.18 | 6.59 | 4.34 | -3.71 |
| pycom08g07690 | WRKY7 | 4.23 | 5.44 | 6.64 | 3.58 | 3.93 | 4.02 | 0.52 |
| pycom09g04600 | WRKY1 | 14.85 | 14.06 | 16.03 | 19.20 | 18.92 | 17.20 | -0.29 |
| pycom09g07040 | WRKY71 | 3.60 | 2.33 | 2.75 | 1.61 | 1.48 | 1.46 | 0.94 |
| pycom09g19270 | -- | 37.89 | 44.84 | 43.03 | 62.08 | 54.36 | 56.92 | -0.45 |
| pycom13g10630 | WRKY75 | 0.45 | 0.27 | 0.19 | 0.00 | 0.15 | 0.00 | #N/A |
| pycom13g21150 | WRKY11 | 26.41 | 23.69 | 23.04 | 12.11 | 15.08 | 15.29 | 0.79 |
| pycom15g09730 | WRKY11 | 10.29 | 9.62 | 9.65 | 11.33 | 10.92 | 11.43 | -0.18 |
| pycom15g25080 | WRKY7 | 2.91 | 3.05 | 3.62 | 6.91 | 4.67 | 6.12 | -0.86 |
| pycom16g05540 | WRKY57 | 23.73 | 25.34 | 26.59 | 20.25 | 19.19 | 17.46 | 0.42 |
| pycom16g12920 | WRKY48 | 2.92 | 3.20 | 3.40 | 3.93 | 3.72 | 3.31 | -0.19 |
| pycom16g20580 | WRKY11 | 42.53 | 38.00 | 48.06 | 34.33 | 37.35 | 32.48 | 0.31 |
| pycom17g22700 | WRKY40 | 0.25 | 0.49 | 0.18 | 0.06 | 0.40 | 0.18 | #N/A |
| pycom02g16120 | AGL6 | 442.46 | 486.97 | 527.05 | 399.41 | 411.51 | 399.06 | 0.28 |
| pycom02g25320 | AGL15 | 0.84 | 0.76 | 1.15 | 0.22 | 0.37 | 0.15 | #N/A |
| pycom13g10590 | FBP2 | 220.83 | 211.67 | 234.35 | 204.21 | 209.05 | 211.18 | 0.10 |

| **Table S7 Transcription level of anthocyanin synthesis structure genes** | | | | | | | |
| --- | --- | --- | --- | --- | --- | --- | --- |
| **Gene ID** | **Gene Name** | **XM-1-fpkm** | **XM-2-fpkm** | **XM-3-fpkm** | **M2-1-fpkm** | **M2-2-fpkm** | **M2-3-fpkm** |
| pycom12g11580 | PcPAL1 | 132.405 | 129.9199 | 128.2716 | 145.5654 | 144.1912 | 141.5007 |
| pycom01g13360 | PcPAL2 | 37.3269 | 36.7681 | 37.7656 | 71.868 | 77.3524 | 83.2253 |
| pycom03g04030 | PcC4H1 | 199.522 | 213.1882 | 201.9839 | 307.3559 | 307.4254 | 290.6121 |
| pycom03g04060 | PcC4H2 | 1.3405 | 0.9727 | 0.6365 | 1.2795 | 1.1439 | 1.0377 |
| pycom03g04040 | PcC4H3 | 0.0304 | 0.2071 | 0.0307 | 0 | 0.0315 | 0.0229 |
| pycom13g22250 | Pc4CL1 | 24.9065 | 24.9439 | 26.6995 | 21.0507 | 21.4947 | 24.1994 |
| pycom17g23190 | Pc4CL2 | 78.8656 | 80.9568 | 83.5174 | 130.4459 | 132.0334 | 128.3123 |
| pycom11g11900 | Pc4CL3 | 48.3705 | 45.0236 | 44.5168 | 190.9196 | 199.9078 | 216.358 |
| pycom02g20960 | Pc4CL4 | 1.5555 | 2.2254 | 1.391 | 1.9613 | 2.3809 | 2.2064 |
| pycom04g00350 | PcCHS1 | 261.8504 | 268.1623 | 251.2476 | 261.8402 | 267.2683 | 269.3549 |
| pycom04g00310 | PcCHS2 | 1115.589 | 1076.916 | 1077.874 | 1288.959 | 1339.39 | 1243.319 |
| pycom420g00730 | PcCHS3 | 15.6805 | 13.1345 | 14.025 | 7.8548 | 9.4194 | 8.319 |
| pycom13g10200 | PcCHS4 | 0.1198 | 0.2038 | 0.1209 | 0.3762 | 0.4965 | 0.2252 |
| pycom07g08120 | PcCHS5 | 0 | 0.08 | 0.0474 | 0.0492 | 0.0974 | 0 |
| pycom01g14550 | PcCHI1 | 67.2942 | 59.3698 | 64.8886 | 76.2195 | 75.7355 | 71.3773 |
| pycom07g17740 | PcCHI2 | 7.8253 | 7.6637 | 6.3692 | 12.9413 | 15.0579 | 12.461 |
| pycom01g18040 | PcCHI3 | 488.0516 | 505.1761 | 515.2905 | 414.1611 | 407.9388 | 397.3208 |
| pycom03g20010 | PcCHI4 | 8.2238 | 5.6769 | 6.461 | 9.7378 | 6.4257 | 6.843 |
| pycom03g21140 | PcFAP1 | 8.3222 | 9.8668 | 8.5949 | 9.3291 | 8.8947 | 7.0957 |
| pycom08g06030 | PcFAP2 | 3.981 | 3.4644 | 3.1394 | 2.6345 | 2.4655 | 3.2689 |
| pycom15g04770 | PcFAP3 | 5.3219 | 4.6423 | 5.7334 | 2.5494 | 3.4114 | 2.781 |
| pycom11g24130 | PcFAP4 | 3.6727 | 3.8785 | 4.4083 | 3.3143 | 2.6901 | 2.4761 |
| pycom15g21590 | PcF3H1 | 455.7332 | 453.5531 | 451.996 | 364.6438 | 371.0995 | 341.4615 |
| pycom02g10320 | PcF3H2 | 313.9019 | 316.5055 | 310.2347 | 264.9242 | 259.8046 | 256.5628 |
| pycom06g17760 | PcF3'H | 130.9506 | 132.1765 | 125.7831 | 81.7097 | 84.1127 | 80.7377 |
| pycom08g20120 | PcDFR | 107.9696 | 110.832 | 105.5514 | 121.8525 | 123.2714 | 118.7316 |
| pycom06g06810 | PcANS | 857.3819 | 849.8846 | 829.4868 | 1331.876 | 1331.291 | 1240.886 |
| pycom07g27990 | PcUFGT | 0.0392 | 0.0333 | 0.1186 | 1.3125 | 1.1367 | 1.4142 |
| pycom06g17760 | PcF3'5'H | 130.9506 | 132.1765 | 125.7831 | 81.7097 | 84.1127 | 80.7377 |
| pycom08g14410 | PcFLS1 | 0.1386 | 0.4717 | 0.0466 | 0.2902 | 0.4309 | 0.1737 |
| pycom15g31720 | PcFLS2 | 284.069 | 279.6663 | 277.4305 | 47.2494 | 46.5355 | 49.0545 |
| pycom16g04240 | PcLAR1 | 20.0323 | 19.1259 | 19.7812 | 45.0631 | 43.2794 | 45.832 |
| pycom13g04100 | PcLAR2 | 3.7792 | 3.2527 | 2.6018 | 1.9548 | 1.7506 | 1.6717 |
| pycom06g18890 | PcLAR3 | 0.2612 | 0.3555 | 0.4744 | 0.2187 | 0 | 0.1179 |
| pycom03g13060 | PcLAR5 | 0 | 0.0874 | 0 | 0 | 0 | 0 |
| pycom15g21590 | PcFHT | 455.7332 | 453.5531 | 451.996 | 364.6438 | 371.0995 | 341.4615 |
| pycom10g26230 | PcANR1 | 133.0724 | 132.7334 | 134.7999 | 65.7069 | 69.7097 | 66.116 |
| pycom05g30700 | PcANR2 | 446.9558 | 442.543 | 467.3273 | 295.3726 | 299.9818 | 319.0983 |

**Table S8 The primers used for RT-qPCR analysis the experiment**

| **Gene ID** | **Gene Name** | **Forward Sequence (5'-3')** | **Reverse Sequence (5'-3')** |
| --- | --- | --- | --- |
| pycom11g07310 | PcTubulin | TGGGCTTTGCTCCTCTTAC | CCTTCGTGCTCATCTTACC |
| pycom12g11580 | PcPAL1 | TCTGCCAGGGAAAGATTATCG | TGAAGTTGAATGGAATGGAATGC |
| pycom01g14550 | PcCHI1 | GAACGGGTGCAAGGAATCTA | AACAGGAGTCCCTCCCAAGT |
| pycom12g11580 | PcCHS2 | GGGTGTACTCTTCGGATTTGG | AAAGGCGGAAACAATACATATACG |
| pycom15g21590 | PcF3H | GGAGAAAGACAAAGTGGAGATAAAGC | ACAAGAAGTGGAAAGGCAAAGTTAC |
| pycom06g06810 | PcANS | AGTTGTTCAGGAAAAGCCAAGAGG | ACAAAGCAGGCAGATAGGAGTAGC |
| pycom15g02070 | PcDFR | ACTGAGGCTGCTGAGGAGAG | TCAAATCCAAGCTGGTAAATGT |
| pycom02g21260 | PcUFGT | GGTCAAACAACTGGCAGTAGAG | TGTCCGCCCGTAGATGGTA |
| pycom09g10130 | PcWER | TCCTGTGACAAAAGAGTCCGTC | CTTCTGTTGTCCATGCTCCCT |
| pycom15g24200 | PcHHO5 | AGCAGCTTGGAGGAACACAA | TTTCGGATGTGAAGGCGGTA |
| pycom16g07230 | PcKUA1 | TTGGCGTTAGAGTCACGGAG | CAGAGGCTGCTCATACTGGG |
| pycom02g26450 | PcbHLH130 | AGGCCGGTAAATGTGAAGGG | ACCATGAGCTTGCTGACCTC |
| pycom06g16970 | PcbHLH062 | AGCAACCACCTGCTGTCTTT | TGTTAGATGTGAGGTGGGCG |
| pycom09g03240 | PcMYB10 | CAGCAGAAGATTTAAGTACGCCATC | TTCTAACAAGGTCTCCCACCAATC |
| pycom05g25770 | PcMYB114 | GCCACATCCGTCATAAGACCTC | GCCACTCATGTGTAACCCTTC |
| pycom09g16830 | PcERF053 | CCAATTCAACAGCAACAGCAAC | CCGACTCTCCGACAAAAGGC |
| pycom17g27080 | PcGSTF12 | GCCACAACTTCAACGACC | CCAGTTTAGCGTCATCAATCA |

**Table S9 FPKM of carotenoid biosynthesis genes**

| **ID** | **Symbol** | **XM-1_fpkm** | **XM-2_fpkm** | **XM-3_fpkm** | **M2-1_fpkm** | **M2-2_fpkm** | **M2-3_fpkm** | **log2FoldChange** |
| --- | --- | --- | --- | --- | --- | --- | --- | --- |
| pycom09g06720 | PSY | 6.9935 | 7.517 | 7.1037 | 14.988 | 15.2271 | 15.4214 | 1.085883 |
| pycom09g18890 | PSY | 19.5539 | 16.1482 | 16.3592 | 21.2512 | 20.1697 | 21.2133 | 0.277245 |
| pycom17g12790 | PSY | 2.9371 | 2.0149 | 2.581 | 0.843 | 0.8835 | 1.318 | -1.2732 |
| pycom04g02050 | PDS | 37.6793 | 34.6684 | 37.9169 | 61.9378 | 67.6783 | 62.1274 | 0.8081 |
| pycom04g19540 | ZDS | 34.4794 | 38.2003 | 35.1203 | 63.31 | 58.6996 | 57.2636 | 0.74056 |
| pycom12g21820 | ZDS | 35.3196 | 35.1586 | 33.8093 | 30.2074 | 30.6029 | 28.4059 | -0.2184 |
| pycom14g07340 | LCY1 | 11.7455 | 10.535 | 13.0318 | 15.6384 | 14.8908 | 15.8766 | 0.40697 |

**A B**


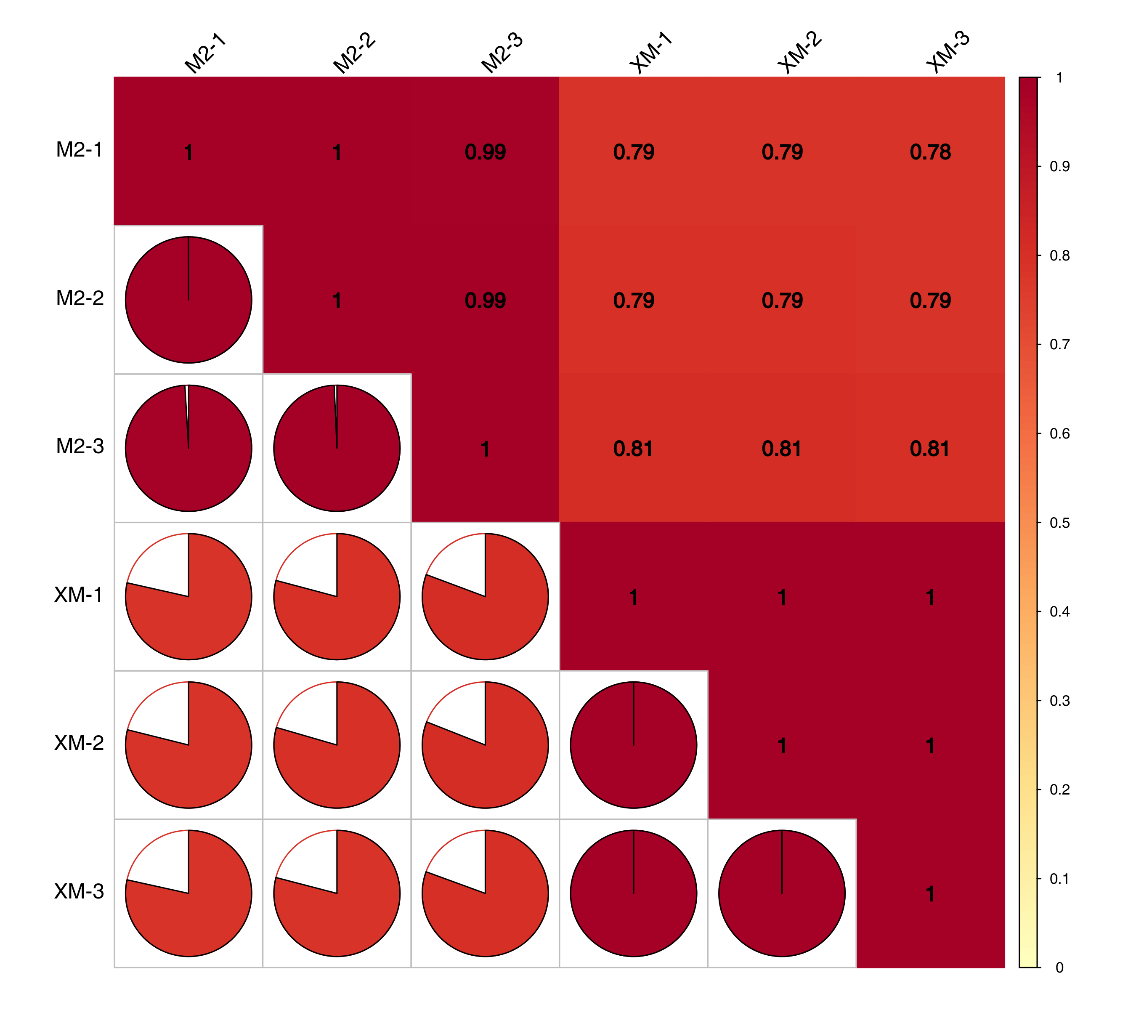

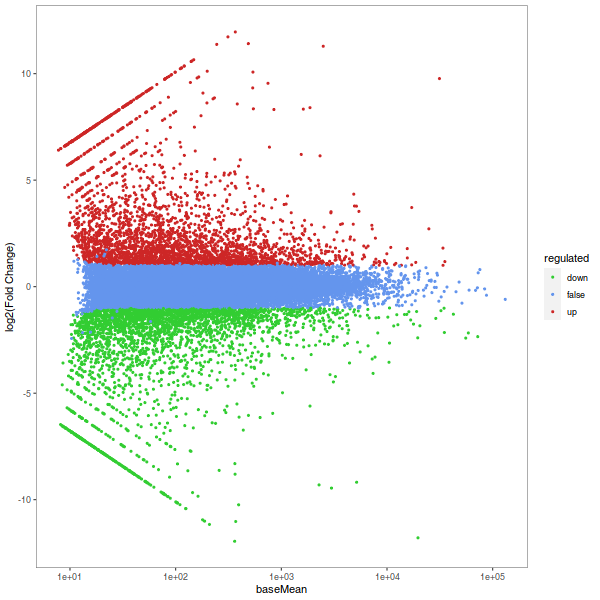


**Figure S1 Transcriptome data used to identify DEGsTranscriptome data used to identify DEGs**

**(A) Sample correlation. (B) Differential gene MA map**

**A**
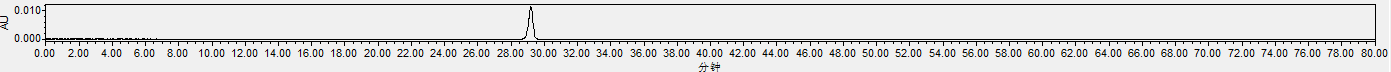


**B**
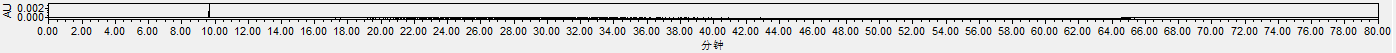


**C**
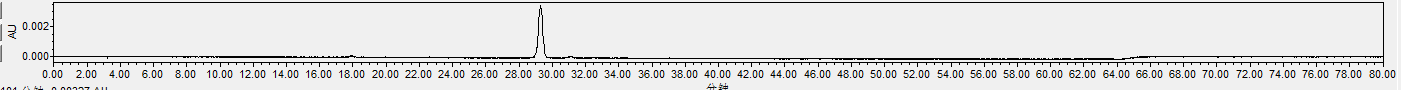


**Figure S2 HPLC spectrogram**

**(A) HPLC spectrogram of standard.** (**B) HPLC spectrogram of ‘XM’.** (**C) HPLC spectrogram of ‘M2’**


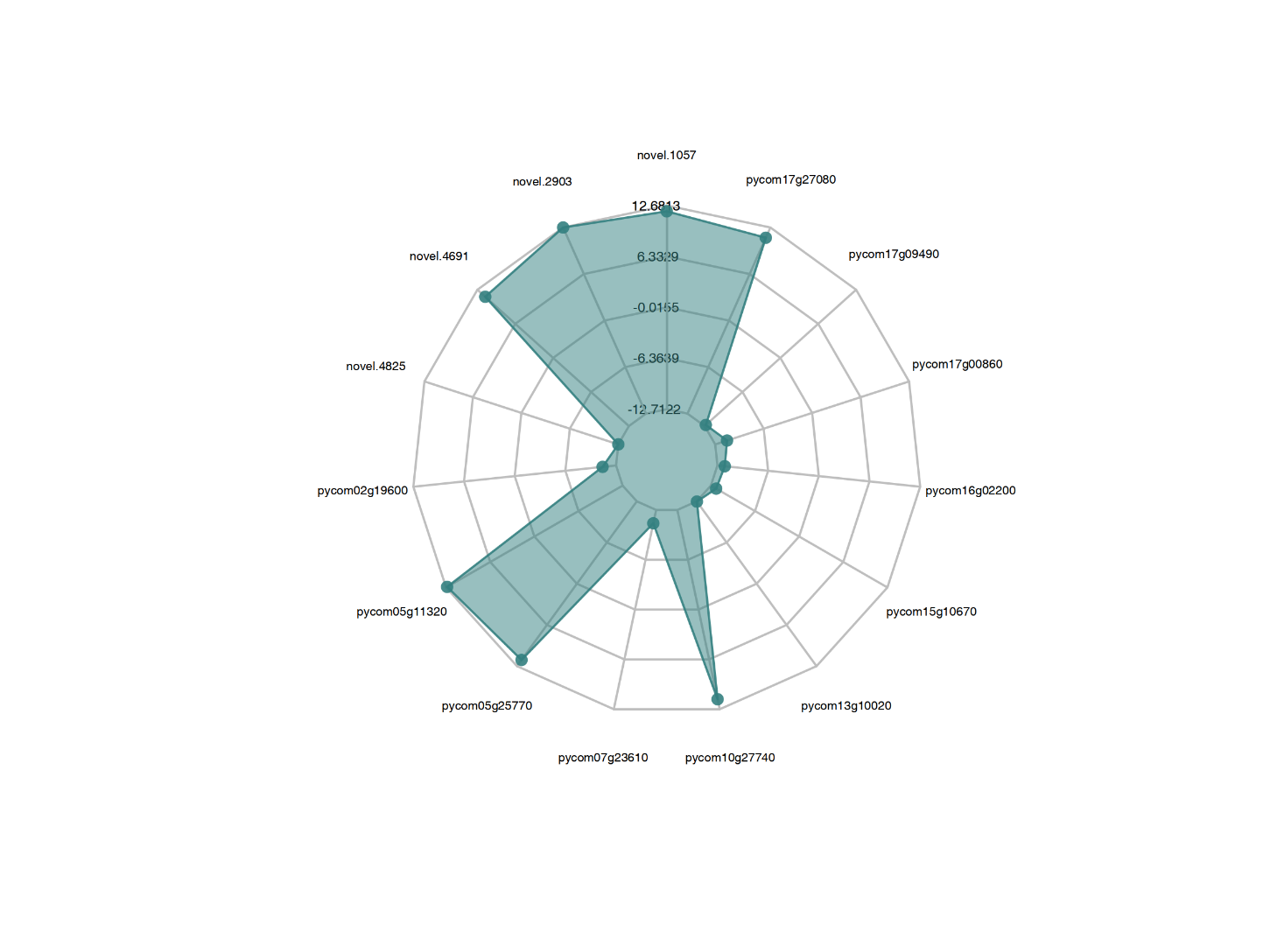


**Figure S3 Top DEGs rader chart**


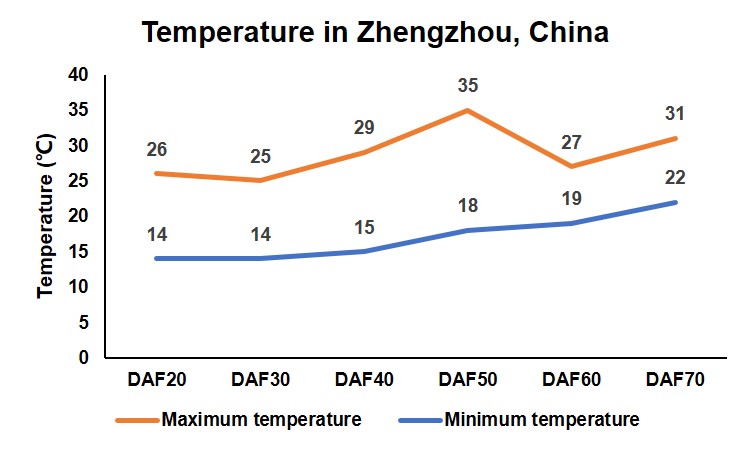


**Figure S4 Temperature in Zhengzhou, China**

**Note: Data sourced from the China Meteorological Administration website (https://data.cma.cn/)**


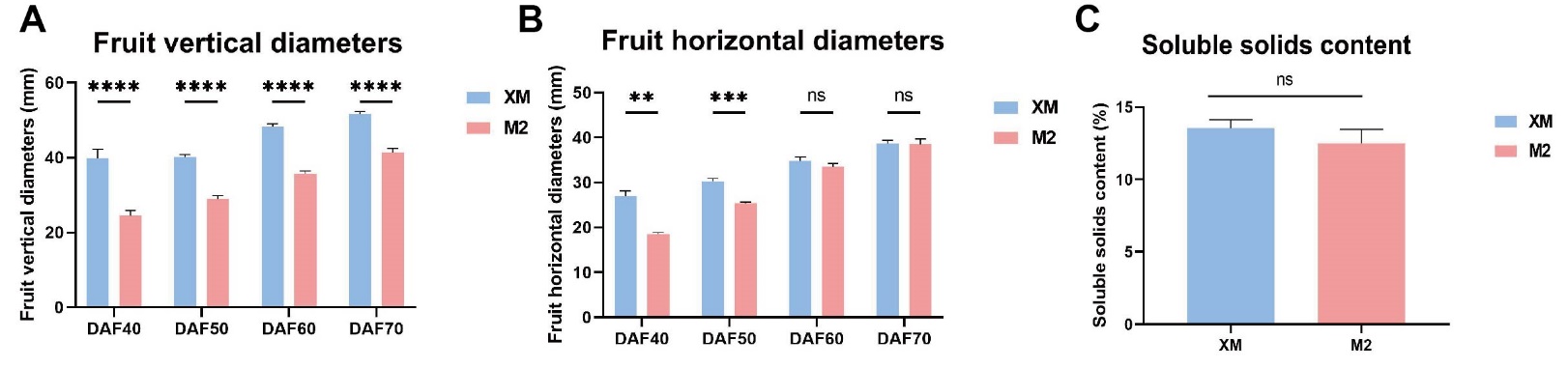


**Figure S5 Fruit quality traits**

**(A) Fruit vertical diameters (B) Fruit horizontal diameters (C) Soluble solids content**

**
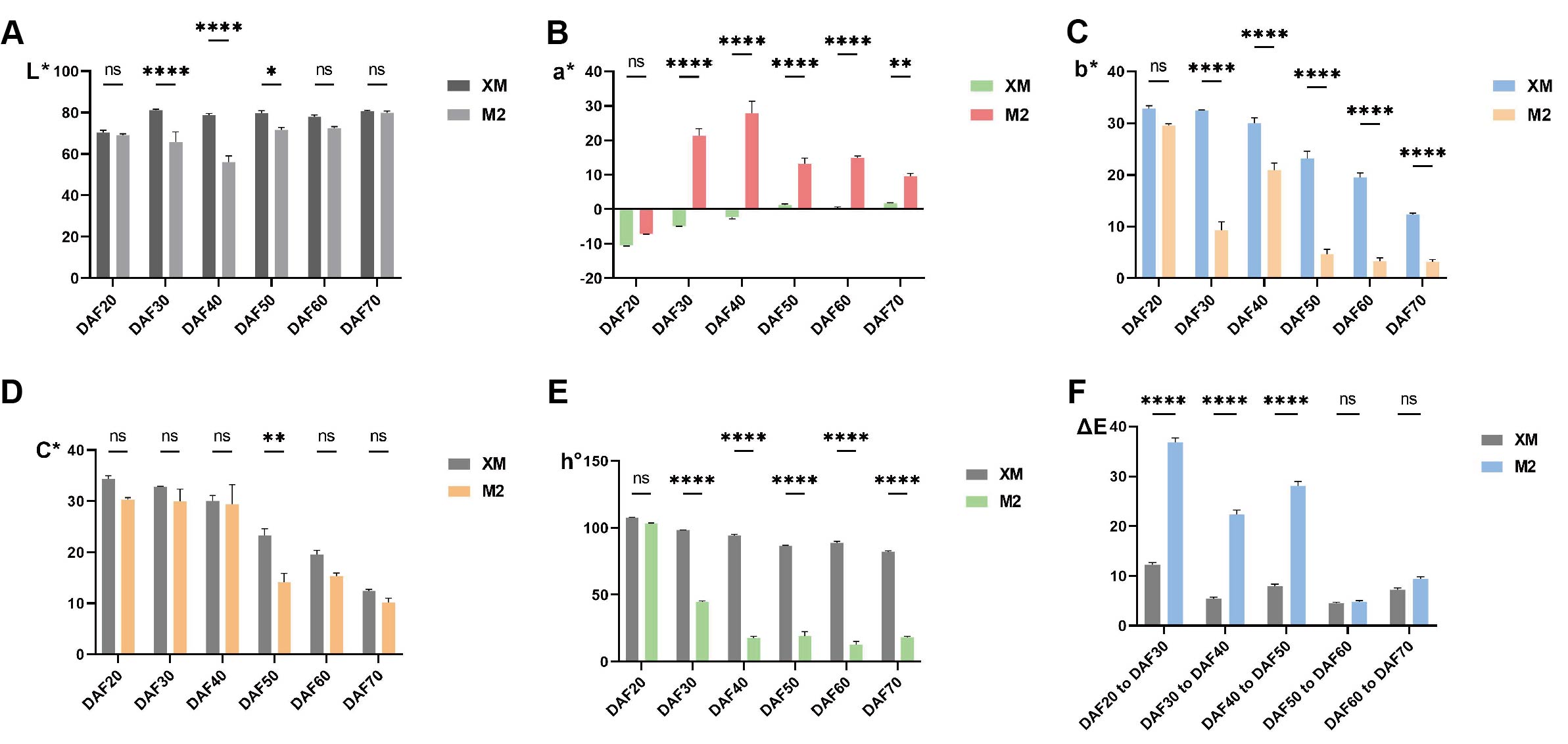
**

**Figure S6 CIELAB color space analysis results**

**(A) Lightness values. (L*) (B)Red-green value(a*). (C) Blue-yellow values (b*). (D)** **Chroma (C*) value. (E)** **hue angle (h°). (F)Total color difference (ΔE).**
